# Taxonomic review of *Tryblionella* with special reference to the Apiculatae group—New characters of genus *Tryblionella* sensu stricto (Bacillariaceae)

**DOI:** 10.1111/jpy.70004

**Published:** 2025-03-18

**Authors:** Rafał M. Olszyński, Ewa Górecka, Rosa Trobajo, Romain Gastineau, Matt Ashworth, David G. Mann

**Affiliations:** ^1^ Faculty of Biology and Environmental Protection, Department of Algology and Mycology University of Lodz Łódź Poland; ^2^ Institute of Marine and Environmental Sciences University of Szczecin Szczecin Poland; ^3^ IRTA‐Institute for Food and Agricultural Research and Technology La Rápita Catalonia Spain; ^4^ Department of Molecular Biosciences University of Texas at Austin Austin Texas USA; ^5^ Royal Botanic Garden Edinburgh Edinburgh, Scotland UK

**Keywords:** Bacillariaceae, diatoms, phylogeny, revision, *Tryblionella*

## Abstract

*Tryblionella* (Bacillariaceae) was described by Smith in 1853, and for many years, the diagnosis of this genus was questionable. Recent molecular analysis based on the *rbc*L gene marker suggests that *Tryblionella* is a polyphyletic genus with *T. apiculata*, *T. hungarica*, and *T. gaoana* forming a distinct group from other *Tryblionella* representatives. Therefore, this study aimed to clarify the diagnosis of *Tryblionella* as a genus. The focus of this study was a selected group of species previously categorized within Grunow's section Apiculatae, which includes the type species *T. acuminata*. This classification serves as a foundation for conducting morphological and molecular comparisons with taxa of *Tryblionella* sensu lato, which are likely to represent distinct and highly diverse genera. Our review includes a detailed examination of frustule ultrastructure and ontogeny combined with a new molecular phylogenetic analysis derived from a three‐gene concatenated dataset. The results of our research indicated that among *Tryblionella*, several monophyletic groups of taxa can be distinguished, including *Tryblionella* sensu stricto (s.s.), by three key characters: a porose valve cross‐section; longitudinal valve undulation, where the peak of the undulation is located on the proximal side of the valve; and the presence of an axial sternum with thickened and relief virgae. *Tryblionella* s.s. taxa share a similar girdle structure: The girdle is graded, except that the first band bears a single row of poroids in the pars exterior and a crenulate margin on the side corresponding to the distal valve mantle.

**Abbreviations**
AU: approximately unbiased test
LM: light microscopy
*psb*C: photosystem II light‐harvesting proteins CP43
*rbc*L: gene‐encoding large subunit of RuBisCO
RuBisCO: ribulose‐1,5‐bisphosphate carboxylase/oxygenase
SEM: scanning electron microscopy
s.l.: sensu lato
s.s.: sensu stricto
SSU: small subunit ribosomal ribonucleic acid

## INTRODUCTION

*Tryblionella* is a genus of raphid diatoms with a characteristically eccentric raphe system, elevated on a keel and supported internally by silica bridges (fibulae) linking its two sides. The original description by Smith (1853) is difficult to interpret (see below), and the genus was reinterpreted by Grunow (1862) and placed in the family “Nitschieae,” now called Bacillariaceae. Subsequently, Grunow refined the classification of the Bacillariaceae (principally in Cleve & Grunow, 1880), and this system was primarily used throughout the 20th century. However, contemporary reassessments are underway, leveraging advancements in morphological analysis and molecular markers facilitated by modern techniques such as scanning electron microscopy (SEM) imaging and the sequencing of selected gene fragments. The changes made so far have mainly improved knowledge of the relationships between certain taxa within the largest and most challenging genus *Nitzschia* (Rimet et al., 2011; Witkowski et al., 2016) and species diversity within *Pseudo‐nitzschia* (e.g., Lundholm et al., 2002), in which multiple species cause harmful algal blooms by domoic acid production (Bates et al., 2018). Nevertheless, molecular phylogenetic and electron microscopical investigations have also been undertaken on *Cylindrotheca* (Li et al., 2007), *Hantzschia* (Maltsev et al., 2021; Souffreau et al., 2013), *Simonsenia* (Kim et al., 2019; Witkowski et al., 2015), and one species belonging to *Tryblionella* (Witkowski et al., 2016). New and available molecular data for Bacillariaceae were reviewed by Mann et al. (2021), indicating the monophyly of some genera (*Bacillaria, Hantzschia, Cylindrotheca, Fragilariopsis, Psammodictyon, Pseudo‐nitzschia*) but not *Nitzschia* or *Tryblionella*. It was suggested that the morphological characters traditionally used for their classification might need reassessment (Mann et al., 2021).

The original description of *Tryblionella* by Smith (1853, p. 35) was as follows: “Frustules free, elliptical or linear; valves plane; alae submarginal or obsolete; canaliculi inconspicuous, parallel.” The genus was contrasted with *Campylodiscus*. Exactly what Smith meant is unclear. He included six species. The “alae” he referred to (literally “wings”) can be equated with the raised raphe system—the “keel” present in many Bacillariaceae and Surirellaceae—while the “canaliculi” (“little channels”) presumably should be elongated, empty tube‐like channels connecting the keel with the interior of the cell, such as exist in *Campylodiscus* and *Surirella* (McLaughlin, 2012; Round et al., 1990). However, such structures are not obvious in the drawings Smith provided nor, indeed, in the species themselves, and Donkin (1869) suggested that Smith was not aware of the real structure of the keel and that his description was inappropriate. Donkin interpreted alae as follows: “…alae are not prolongations of the margin, nor placed at the margin, but are produced by the *abrupt inflection* or folding inwards of the sides of the valve…” (p. 290).

For the next few years, *Tryblionella* was considered a separate taxonomic entity from *Nitzschia*. In 1862, Grunow revised *Tryblionella*, adding some species but excluding some of Smith's. At that time, he separated *Tryblionella* from *Nitzschia* by the absence of fibulae or their scarce visibility: “hauptsächlich durch den Mangel der Kielpunkte (oder nur schwache Andeutungen derselben)” (p. 551), whereas, as we now know, they are present in both genera. He added that *Tryblionella* valves usually have a longitudinally running fold. However, later, based on his and Kitton's observations, Grunow (1878) decided that the visibility (or absence) of fibulae and longitudinal undulation did not provide any basis for considering *Tryblionella* a genus; therefore (in Cleve & Grunow, 1880), he assigned all known *Tryblionella* species to different groups within *Nitzschia*. These were characterized by an eccentric raphe system and indistinct fibulae and separated by the shape of the valve (in some cases with a constriction in the middle of the valve) and the presence or absence of a longitudinal interruption of the striae (Cleve & Grunow, 1880; Grunow, 1879). Subsequently, Grunow's groups—Tryblionella, Apiculatae, Pseudotryblionella, Circumsutae*—*were treated as sections of *Nitzschia*, but with time, all were combined again by Hustedt (1930) within one group: sect. *Tryblionella*. This group was characterized by a strongly eccentric keel and the presence of a more or less clearly visible longitudinal fold, in which there was often an interruption to the striae. For the first time, it was noted that the species belonging to this group occurred predominantly in saltwater environments.

The introduction of electron microscopy provided the opportunity to undertake more detailed studies of frustule morphology. Mann (1978) used SEM, which provided a more comprehensive examination of the morphological structure within the section *Tryblionella* sensu Hustedt than had been previously described and focused on two main groups of species. One of these contained *Nitzschia tryblionella*, *N. debilis*, and *N. circumsuta*, and the other *N. apiculata*, *N. hungarica*, and *N. acuminata*. These two groups differed in valve shape, patterns of undulation and areolation, and fibula density, as well as the presence of an axial sternum and the development of a marginal ridge. Twelve years later, Mann (in Round et al., 1990) restored the genus *Tryblionella*, defining it polythetically, using combinations of characters including an undulate valve, interrupted striae, squat fibulae (which may be porous), and a marginal ridge on the proximal edge. In the same work, a new genus *Psammodictyon* was described for a group of species originally also considered to be within or closely related to *Tryblionella* but possessing chambered pores (loculate areolae) arranged in decussate rows.

A recent molecular phylogenetic study of the Bacillariaceae (Mann et al., 2021) did not resolve a monophyletic *Tryblionella*, since *T. debilis*, the *T. apiculata* group (*T. apiculata*, *T. hungarica*, and *T. gaoana*), and *T. compressa* occurred in three different clades. Mann et al. (2021) identified some morphological differences between these molecular clades; for example, *T. debilis* had shallow, hollow, and striated fibulae, and the *T. apiculata* groups had small, solid fibulae. However, one barrier to progress remains the poor taxon sampling for molecular phylogenetics and patchy morphological information. The aim of this study was, therefore, to clarify the diagnosis of *Tryblionella* as a genus and focus on a group of species that were included in Grunow's section Apiculatae, which contains the type of *Tryblionella*, *T. acuminata*, and provides a basis for comparisons with other groups of *Tryblionella* sensu lato (s.l) taxa. Our review has included a detailed examination of frustule ultrastructure, as well as thorough light microscopy (LM) based investigations, combined with a new molecular phylogenetic analysis derived from a three‐gene concatenated dataset (*rbc*L, *psb*C, and SSU rRNA gene) and phylogenetic analysis of published morphological data derived from electron microscopy of *Tryblionella* s.l. and *Nitzschia* s.l. taxa.

## MATERIALS AND METHODS

For the purposes of this study, a total of 31 taxa of *Tryblionella* s.l. and *Nitzschia* s.l. were analyzed. Among these, eight strains of the family Bacillariaceae were investigated based on morphological characteristics and molecular markers that have also been included in previous research. Try986CAT—*T. hungarica*, Try981CAT—*T. hungarica*, Try946CAT—*T. apiculata*, s0863—*T. apiculata* (Mann et al., 2021), FLMan33 panduriformA23—*T. apiculata* (Frankovich et al., 2018), BC0502—*T. debilis* (Mann et al., 2021), TA426—*Nitzschia gaoana* (An et al., 2017), and Tokiane4 nitzG1—*Nitzschia* sp. (Lobban et al., 2021). Four new strains were isolated from samples collected in Poland (Pełczyska salty pond: SZCZ E683—*Tryblionella hungarica* and the Baltic Sea: SZCZ E2191—*T. hungarica*) and Palau in Micronesia (Rebotel Reef: RebReef3_elongpeanutPDK142—*T. marginulata* and RebReef2_pandurifPDK38—*T. marginulata*). Morphological investigation was conducted on *T. navicularis* from a brackish‐marine sediment sample from Ferrybridge, Dorset, England, collected c. 1977 (voucher slide A108 in the diatom herbarium, Royal Botanic Garden Edinburgh) and *T. plana* var. *fennica* from an environmental sample. A list of analyzed strains, taxa, and sampling locations is included in Table S1 (Bertolli et al., 2020; Cavalcante et al., 2013; Guttinger, 1989; Im et al., 2020; Lee et al., 2023; Mann, 1984; Round & Basson, 1997; Witkowski et al., 2004).

The Pełczyska benthic and water samples were collected from the silty shore of Pełczyska pond in Pełczyska village (Łódź Voivodeship, 51°58′35.68″ N; 19°14′17.02″ E) in 2014 (October and December) and 2015 (March and July). The method of the analysis of chemical parameters was described by Żelazna‐Wieczorek et al. (2015). Environmental conditions for the Rebotel Reef collections were published by Hoadley et al. (2019). The Baltic Sea planktonic sample was collected from the Baltic Sea littoral near Niechorze village (West Pomeranian Voivodeship, 54°5′59.17″ N; 15°5′28.72″ E) in June 2022. Analysis of water parameters in situ pH, temperature, and salinity was performed using a multiparameter meter Hach HQ series (Hach, Loveland, Colorado, United States). Sandy sediment in the littoral of the Lake of Menteith, Stirlingshire, Scotland (56°10′51″ N, 4°17′3.5″ W) was sampled from c. 1 m depth among reeds on 29 September 2005. Epipelon containing *Tryblionella plana* var. *fennica* was harvested from the sediment using the method described by Mann et al. (2008); unmounted material and voucher slides are kept in the Royal Botanic Garden Edinburgh (E), accession E3588.

In Pełczyska pond, the environment was characterized by alkaline water (pH > 9) and high electric conductivity (up to 5150 μS · cm^−1^). The concentration of chloride (up to 1524 mg · L^−1^), potassium (up to 69 mg · L^−1^), phosphates (up to 4.52 mg · L^−1^), and sodium ions (up to 689 mg · L^−1^) were high (Żelazna‐Wieczorek et al., 2015; Żelazna‐Wieczorek & Olszyński, 2016). Baltic Sea water was characterized by low salinity (7.1) and slightly alkaline (pH = 8.0). Values of all tested parameters are given in Table S2.

**FIGURE 1:**
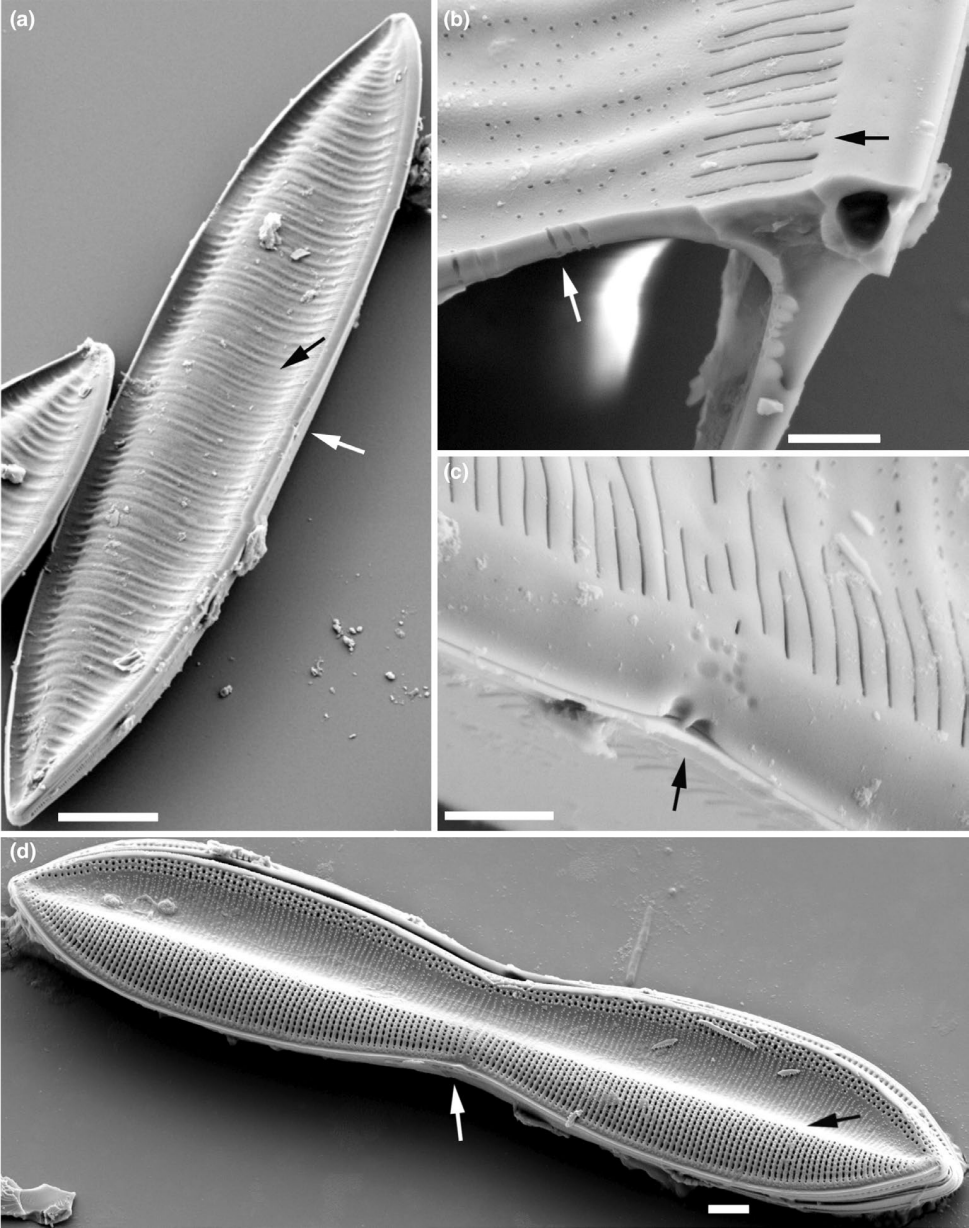
Scanning electron micrographs illustrating the characteristics of the analyzed *Tryblionella* s.l. taxa. (a–c) *Tryblionella gracilis*, (d) *T. marginulata*. (a) The longitudinal valve undulation with the depression (black arrow) located on the proximal side of the valve (white arrow). (b) Cross‐section of the valve face with a porous structure, showing areolae through the silica layer (white arrow). Areolae on the raphe canal merging into slits (black arrow). (c) The center of the external part of the valve has slightly deflected proximal raphe fissures (arrow). (d) The longitudinal valve undulation with the peak (black arrow) located on the proximal side of the valve (white arrow). Scale bars = 10 μm (a), 2 μm (d) or 1 μm (b, c).

**FIGURE 2:**
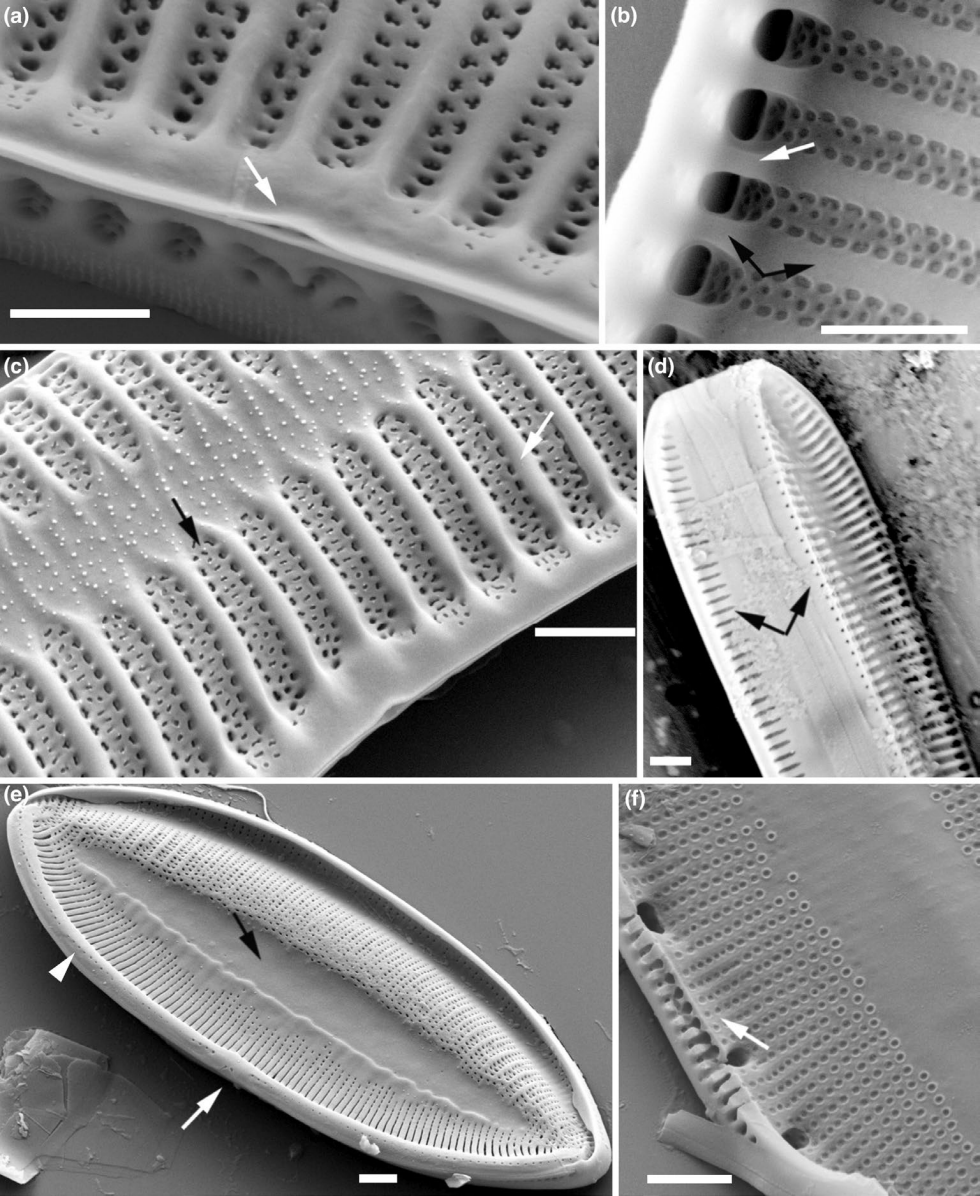
Scanning electron micrographs illustrating the characteristics of the analyzed *Tryblionella* s.l. taxa. (a) *Tryblionella hungarica*, isolate TRY981CAT, (b, c) *T. apiculata*, isolate TRY946CAT, (d) *T. hungarica*, isolate SZCZ E683, (e–f) *T. debilis*, isolate BC0502. (a) Proximal raphe fissures, straight and nested in a notch (arrow). (b) The interior view shows one solid fibula without poration (white arrow), corresponding to one virga (black arrows). (c) The external view of the valve face shows striae composed of three rows of areolae, which extend along the raphe canal tube (black arrow). Note that the structure of the areole toward the raphe canal is fixed and unchanging. Some virgae are thickened as much more prominent ribs (white arrow). (d) The girdle view illustrates the shape and size differences of the areolae on the proximal and distal mantle (arrows). (e) Valve view with an axial sternum and lacking any distinct features (black arrow). Proximal raphe fissures are distally directed (white arrow). The arrowhead indicates areolae, or pits that appear to be areolae and are present on the raphe canal tube. (f) The internal view shows a wide and perforated fibula corresponding to several virgae (arrow). Scale bars = 1 μm, except (d) = 2 μm.

**FIGURE 3:**
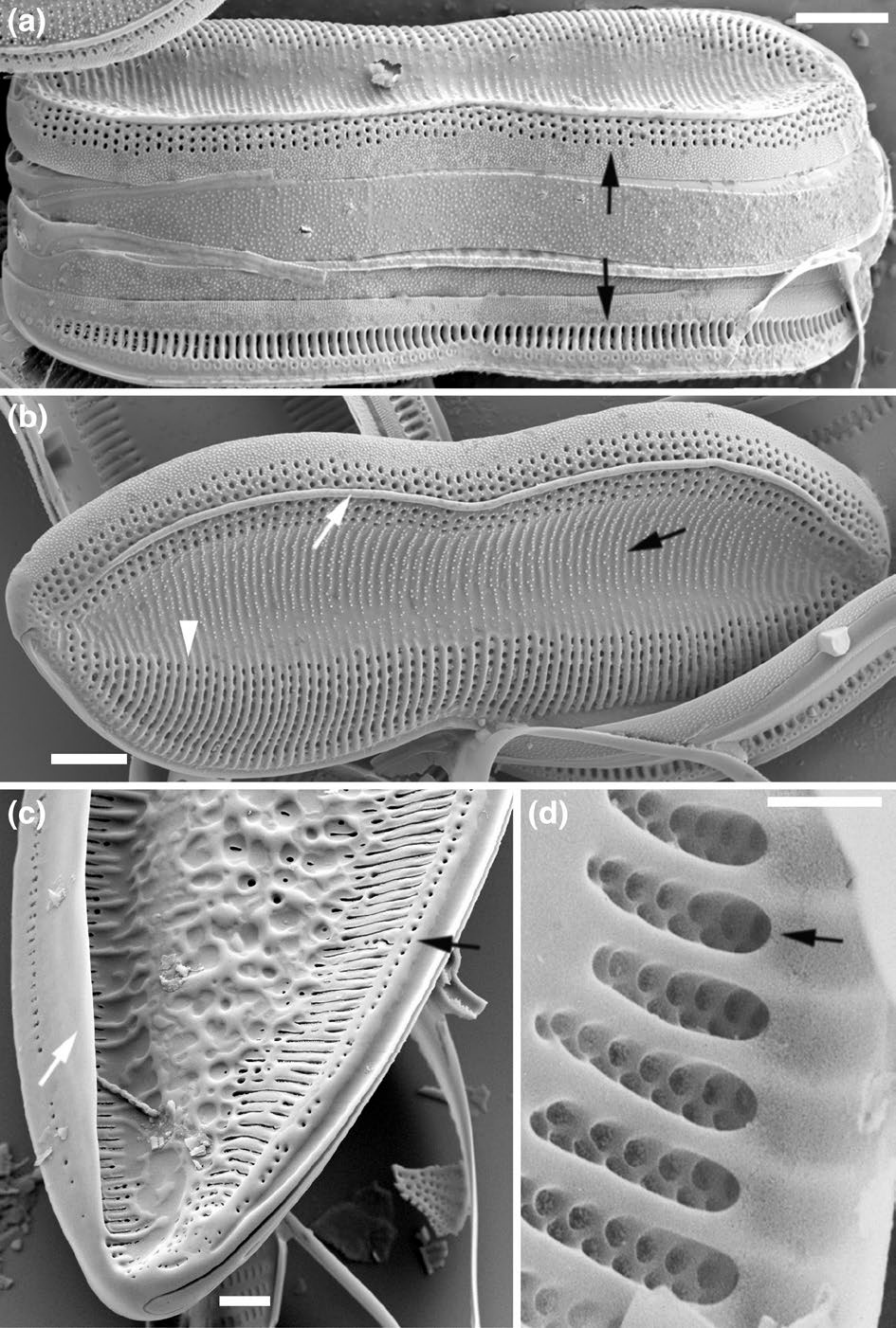
Scanning electron micrographs illustrating the characteristics of the analyzed *Tryblionella* s.l. taxa. (a, b) *Tryblionella marginulata*, (c) *T. plana* var. *fennica*, (d) *T. navicularis*. (a) The girdle view illustrates the differences in shape, size, and density of the areolae on the proximal (bottom) and distal (top) mantle (black arrows). The external view of the valve face and mantle. A white arrow indicates a marginal ridge as a short, narrow cylinder of silica (piping). Axial sternum with visible papillae and thickened virgae (black arrow). Striae are composed of one row of areolae (arrowhead). (c) Frustule pole. White arrow shows a sail‐like marginal ridge. Look at the areolae located on the raphe canal (black arrow). (d) Alveolate cross‐section with an alveolus (arrow). Scale bars = 2 μm.

A dataset of morphological characters was constructed based on data derived from SEM images obtained from the strains listed above, supplemented by taxa with published SEMs listed in Table S1. To find published SEMs, we searched for manuscripts with images of either *Tryblionella* or Bacillariales taxa with longitudinally undulate valves like *Tryblionella*. The characters chosen for the phylogenetic analysis are listed in Table 1. The dataset was analyzed using TnT ver. 1.6 (Goloboff & Morales, 2023) by a New Technology Search using 100 random addition sequences with 50 ratchet substitutions and the “collapse trees after search” option enabled. All other parameters were left at the default setting and *Psammodictyon constrictum* was set as the outgroup. Figures 1, 2, 3 and Table 1 provide examples, a list, and descriptions of the analyzed characters.

**TABLE 1:** Analyzed morphological characters of *Nitzschia* s.l. and *Tryblionella* s.l.

Character	Character states			
(1) Valve cross‐section	0. Porose – a valve structure in which areolae are not bound in any three‐dimensional structure. The delimitation of pores is achieved by fusing vimines between virgae (Cox, 2012) Figure 1b	1. Pseudoloculate – According to Ross et al. (1979): chamber formed on the outer side of the basal siliceous layer by expansion of the distal parts of anastomosing of reticulate costae	2. Alveolate – a valve structure that consists of an alveolus – an elongated chamber containing areolae, opened to the interior of the frustule (Ross et al., 1979) Figure 3d	
(2) Longitudinal valve undulation	0. undulation absent – the valve face is predominantly flat	1. Nadir at raphe – the undulation depression is located on the proximal side of the valve Figure 1a	2. Peak at raphe – the peak of the undulation is on the proximal side of the valve Figure 1d	
(3) Axial sternum – a longitudinal part of the valve where areolae are scattered or absent. It is located centrally as the axial sternum. The sternum separates the areolae on the valve face (Chiappino et al., 1977; Mann, 1978; Round et al., 1990)	0. axial sternum absent or indistinct	1. Axial sternum present and featureless. No visible, relief virgae within sternum Figure 2e	2. Axial sternum present with papillae and/or thickened, and relief virgae present within the sternum Figure 3b	
(4) Marginal ridge	0. marginal ridge absent or indistinct	1. Present non‐continuous – ridge is present, but interrupted or discontinuous, that is, *T. victoriae* in Bertolli et al. (2020) figure 11	2. Piping – ridge is present as a short, narrow cylinder of silica Figure 3b	3. Sail‐like – ridge is relatively high, forming a thin extension of the mantle perpendicular to the valve face Figure 3c
(5) Stria	0. Striae are uniseriate Figure 3b	1. Striae are bi‐ or multiseriate Figure 2c		
(6) Virga thickening	0. Equal thickness – the virgae are almost all the same thickness and more or less the same thickness as the frets (vimines), that is, *T. circumsuta* in Bertolli et al. (2020) Figure 6a	1. Some thickened – some virgae, or perhaps some “groups of” virgae, are thickened as much more prominent ribs Figure 2c		
(7) Virgae to fibulae ratio	0. One virga corresponding to one fibula Figure 2b	1. More than one virga corresponds to one fibula Figure 2f		
(8) Fibulae type	0. Solid – fibulae present without any ornamentation or perforation Figure 2b	1. Perforated – fibulae present with poroids Figure 2f		
(9) Central raphe endings	0. Central raphe endings absent or indistinct	1. Present, straight and nested in a notch Figure 2a	2. Present, slightly deflected Figure 1c	3. Present, with distally directed fissures Figure 2e or *T. circumsuta* in Bertolli et al. (2020) figure 6d
(10) Stria structure at the raphe canal	0. No change Figure 2c	1. Areolae merging externally to slits Figure 1b	2. Striae become biseriate that is, *T. compressa* in Mann and Trobajo (in press) figure 18	
(11) Areolae on the raphe canal	0. No areolae present (or areolae indistinct) on the raphe canal tube	2. Areolae, or pits which appear to be areolae, present on raphe canal tube Figures 2c,e, 3c		
(12) Mantle areolae	0. The density, shape and size of the mantle areolae are the same on both the raphe and rapheless mantle	1. The shape and size of the mantle areolae differ between the raphe and rapheless mantle Figure 3a	2. The density of the mantle areolae differs between the raphe and rapheless mantle Figure 2d	

Methods of culture, DNA extraction, and LM and SEM preparation from culture and environmental samples are provided in Appendix S1. The lists of sequences with corresponding accession numbers, as well as the alignments used for phylogenetic analysis, are available at https://doi.org/10.5281/zenodo.10997217. Methods of sample preparation for LM and SEM analysis, DNA extraction, and sequencing of the Tokiane4 and Palau strains follow the protocols described in Ashworth et al. (2017) and Lobban et al. (2021); for the various Try…CAT and s0863 strains, see Mann et al. (2021).

### Regarding the terminology of the sternum

Mann introduced the term “sternum” in his doctoral dissertation (1978). He characterized the sternum as an elongated section of the valve where areolae are sparse or absent relative to the remainder of the valve and which is often thickened pervalvarly. His research delineated various types of sterna based on their positioning on the valve: *the raphe sternum*, which is located on both sides of (i.e., surrounds) the raphe, which may be centrally positioned, as exemplified by *Navicula* species, or marginally located, as observed in *Nitzschia* species; *the axial sternum*, which lacks a raphe but is positioned along the central axis of the valve; and *the lateral sternum* (which also lacks a raphe), which is shifted toward the edge of the valve, as seen in *Tryblionella debilis*. Subsequent studies have increasingly associated the term raphe sternum with the raphe of the ribs, which represent the early developmental stages of the valve (Chiappino et al., 1977; Chiappino & Volcani, 1977; Round et al., 1990). For the purposes of this study, we recognized the specific classifications of the raphe, axial, and marginal (in place of >lateral sterna>) sterna as longitudinal zones of the valve where areolae are either dispersed or absent.

## RESULTS

### Morphological description

For brevity and to focus attention on key issues relating to *Tryblionella* systematics, we have presented the primary morphological data on each species in the supplemenatry figures. These data provide the basis for the character matrix used in the “Morphological analysis” below. The supplemtary figures [Link], [Link], [Link], [Link], [Link], [Link], [Link], [Link], [Link], [Link] include plates accompanied by captions that provide a comprehensive description of the morphological characteristics and detail the findings related to the analyzed strain morphology for *T. apiculata* (Figures [Link], [Link]), *T. hungarica* (Figures [Link], [Link]), *T. marginulata* (Figure S6), *T. gaoana* (Figures [Link], [Link]), *T*. cf. *gaoana* (Figure S8), and *T. plana* var. *fennica* (Figure S9). Additionally, we have included a plate with the photos of *T. acuminata* (Figure S10) used by Mann (1978), reworked from the film negatives.

### Valve ontogeny

**FIGURE 4:**
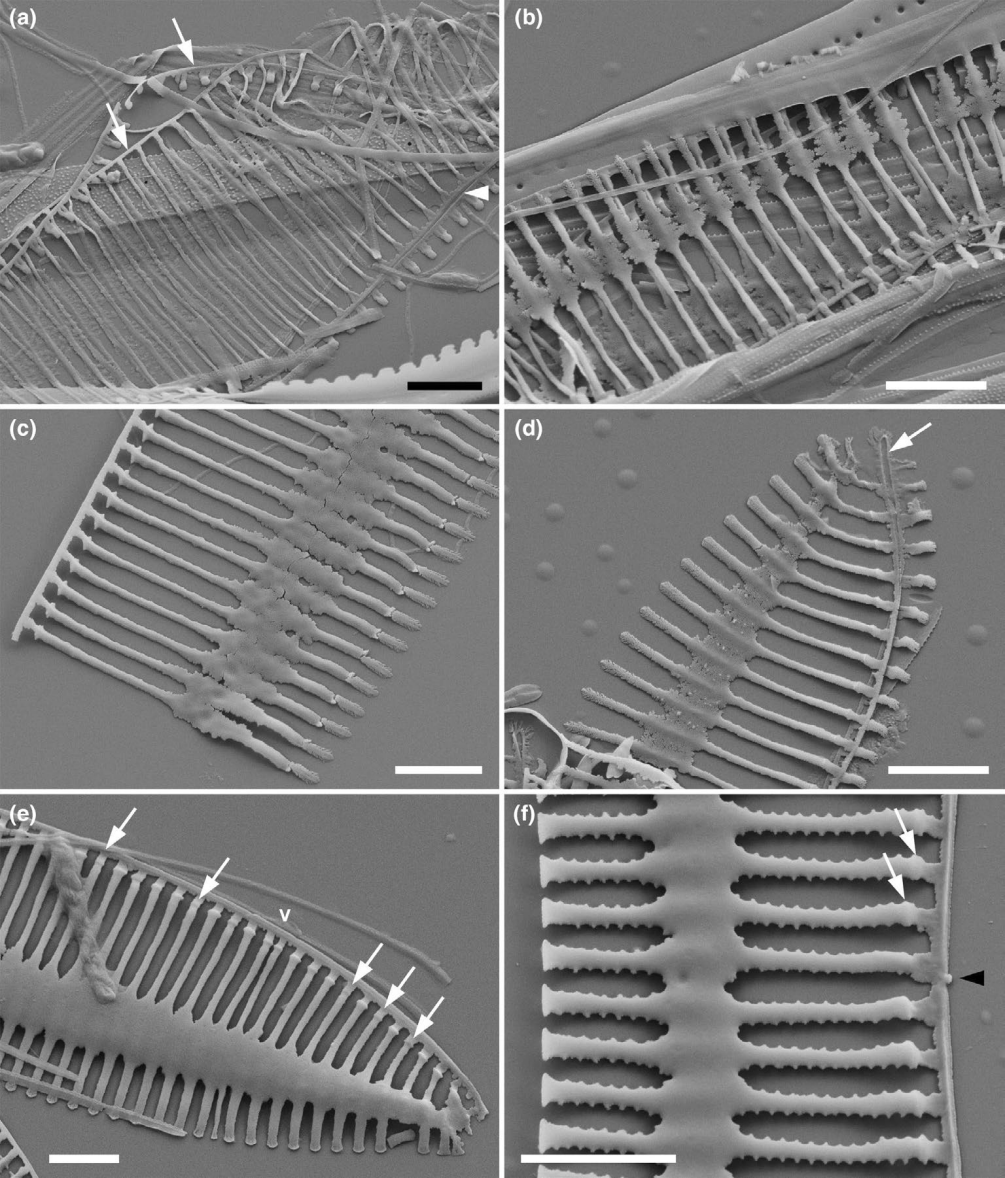
*Tryblionella* spp., stages in valve development, SEM. (a–c). *Tryblionella apiculata*, isolate TRY946CAT, (d–f). *T. hungarica*, isolate TRY981CAT. (a) Early stage in which the transapical ribs appear as delicate strings connected only by the raphe sternum. Two sibling valves are superimposed here: In one, the two sides of the raphe sternum have sprung apart (arrows); in the other, the raphe is intact (arrowhead), separating the wide valve face from the narrow mantle. (b) Transapical ribs almost fully extended, with the beginnings of axial sternum formation. (c) Valve face exterior (the proximal mantle has broken off): The axial sternum is almost complete, and the raphe canal is beginning to be delimited. (d) Slightly later stage, in which the axial sternum is complete but not thickened. The raphe slit (arrow) separates the valve face from the much narrower (proximal) mantle. (e) Valve face interior (the proximal mantle has broken off): stage very slightly earlier than that shown in (f). Small knobs are present on the ribs near the raphe (e.g., arrows) and will develop into the fibulae. A tiny projection (arrowhead) marks where the forming valve has split at the central raphe endings. Small knobs are forming close to the raphe, but not on every rib: Those ribs lacking knobs (e.g., arrows) will not develop fibulae. At “v,” two more closely spaced ribs mark a Voigt discontinuity. (f) A forming valve that has split along the raphe, leaving the valve face without the corresponding mantle: interior view. The axial sternum and transapical ribs are almost fully formed and small spiny outgrowths from the ribs indicate the beginnings of areola formation. Scale bars = 2 μm.

In the Ebro Delta clones Try946CAT and Try981CAT, representing *Tryblionella apiculata* and *T. hungarica*, respectively, stages of valve formation were frequent, making it possible to determine when the fibulae and axial sternum were formed relative to other features, such as the raphe system and areolae (Figure 4). In the earliest stage observed (Figure 4a), valves consisted of an extremely narrow raphe sternum, accompanied by equally narrow transverse ribs on each side. On the proximal side, the ribs were extremely short, leading to the shallow nature of the proximal valve mantle in fully formed valves. No trace of an axial sternum, fibulae, or areolae were present at this stage. Slightly later (Figure 4b), the axial sternum began to form through feathery outgrowths from the ribs. As outgrowths extended further, they came into contact and began to fuse (Figure 4c), finally creating a continuous plain band along the valve (Figure 4d). At this stage, there were still no fibulae, and no areolae had been delimited. A row of small lateral spines developed close to the raphe (Figure 4c), demarcating the future raphe canal from the remainder of the valve face. In addition, small knob‐like thickenings began to appear along the line of demarcation (Figure 4e). In *T. apiculata*, each transapical rib (apart from one or two at the center) bore a thickening (Figure 4f), whereas in *T. hungarica* some of the ribs remained without thickenings (Figure 4e, arrows). The knob‐like thickenings were incipient fibulae and subsequently extended across beneath the raphe to create the robust bridges present in the mature valves (cf. Figures S1b, S4b,c). Areolae began to be formed simultaneously with the fibulae, those closest to the transapical ribs (virgae) being formed first (Figure 4f). Until the fibulae were complete, the forming valves remained liable to break longitudinally at the center because of the extremely small size of the “central nodule” connecting the two sides of the raphe sternum (Figure 4f). The fact that the transapical ribs (virgae) are formed across the whole width of the valve before any of them extend laterally and fuse to form the axial sternum was sometimes reflected in Voigt discontinuities that likewise traversed the whole width of the valve (Figure 4e).

**FIGURE 5:**
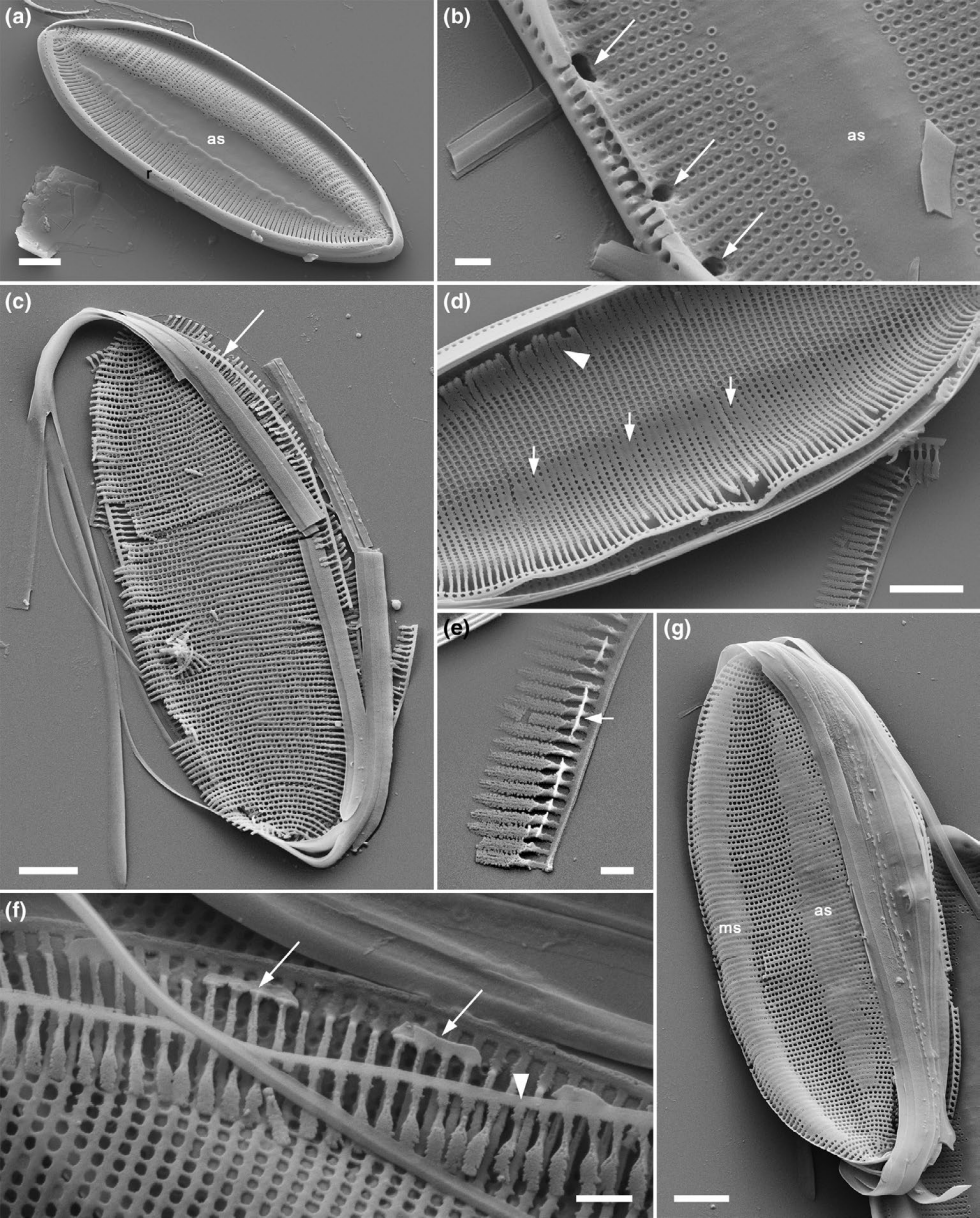
*Tryblionella debilis*: Ultrastructure and stages in valve formation, SEM. (a) Mature valve, exterior. The raphe (**r**) runs along the nearer side in this photograph; on the opposite side, there is a strongly developed marginal ridge. Along the midline of the folded valve face the striae are interrupted by a plain band, the axial sternum (**as**). (b) A mature valve, in which part of the proximal mantle has been broken off revealing the structure of the fibulae, which are wide structures separated by small round or elliptical interspaces (arrows). Note that the valve face striae continue onto and across the fibulae. In the axial sternum (**as**) there is scarcely any hint of transapical ribbing or poroids. (c) Ontogenetic stage (surrounded by girdle bands from the parental frustule), with transapical ribs and striae extending across the whole width of the new valve. No sterna are present. The raphe sternum is located on the right (e.g., arrow). (d) Slightly later stage, in which poroids along the midline (arrows) and toward the distal mantle (arrowhead) are beginning to be filled in to form the axial and marginal sterna. (e) Fragment of a partially formed proximal mantle (detail of (d)): Projections have developed on some of the transapical ribs of the mantle (e.g., arrow) and are fusing laterally in the first stages of fibula formation. (f) Detail of the forming raphe sternum, which has split apart during specimen preparation: On the proximal mantle (arrowhead) the transapical ribs (virgae) have yet to become interconnected. On the valve face, a well‐developed system of ribs and poroids is present and the fibulae are beginning to form (arrows). (g) Later stage, in which the axial and marginal sterna (**as** and **ms**) are clearly visible though still incomplete. Note that, in (f) and (g), the fibulae are separated from the raphe by a single line of poroids. Scale bars = 2 μm (a, c–d, g) or 0.5 μm (b, e–f).

Valve ontogeny was also examined in *Tryblionella debilis* (Figure 5) for comparison with *T. hungarica* and *T. apiculata* (Figure 4) because of the polyphyletic position of *T. debilis* relative to these other taxa according to genetic data (see below). In the mature valves of *T. debilis*, the striae are interrupted by two plain areas, one along the center of the valve (forming an axial sternum), as in the other *Tryblionella* species described here, and the other close to the distal margin (forming a marginal sternum; Figure 5a,b). The striae are single rows of small round areolae (Figure 5b). Unusually for the Bacillariaceae, the striae of *T. debilis* valves continue into and across the fibulae, which are very wide relative to the circular or elliptical interspaces between them (Figure 5b).

In the first ontogenetic stages observed in *Tryblionella debilis*, the transapical ribs (virgae) extended across the whole width of the valve (Figure 5c), but in contrast to *T. hungarica* (Figure 4d), areolae were almost fully delimited throughout, with no indication of axial or marginal sterna (contrast Figure 5c). Subsequently, areolae were progressively filled in along the midline of the valve and near the distal margin (Figure 5d,g), so that scarcely any trace of them was visible in the mature valve (Figure 5b). The raphe sternum was very narrow throughout, so that before the fibulae were completed, the valves usually split apart along the line of the raphe (Figure 5c,f), as in *T. hungarica* and *T. apiculata*. The fibulae were initiated from the transapical ribs, initially as a series of separate projections, which rapidly coalesced into elongated flanges (Figure 5e,f). Considering the process of ontogenesis and the timing of the development of the axial sternum, it can be established that the *primary axial sternum* manifests prior to the formation of areoles and the fibula. In contrast, the *secondary axial sternum* develops subsequently to the appearance of these structures.

### Morphology of the girdle bands

#### Tryblionella hungarica

**FIGURE 6:**
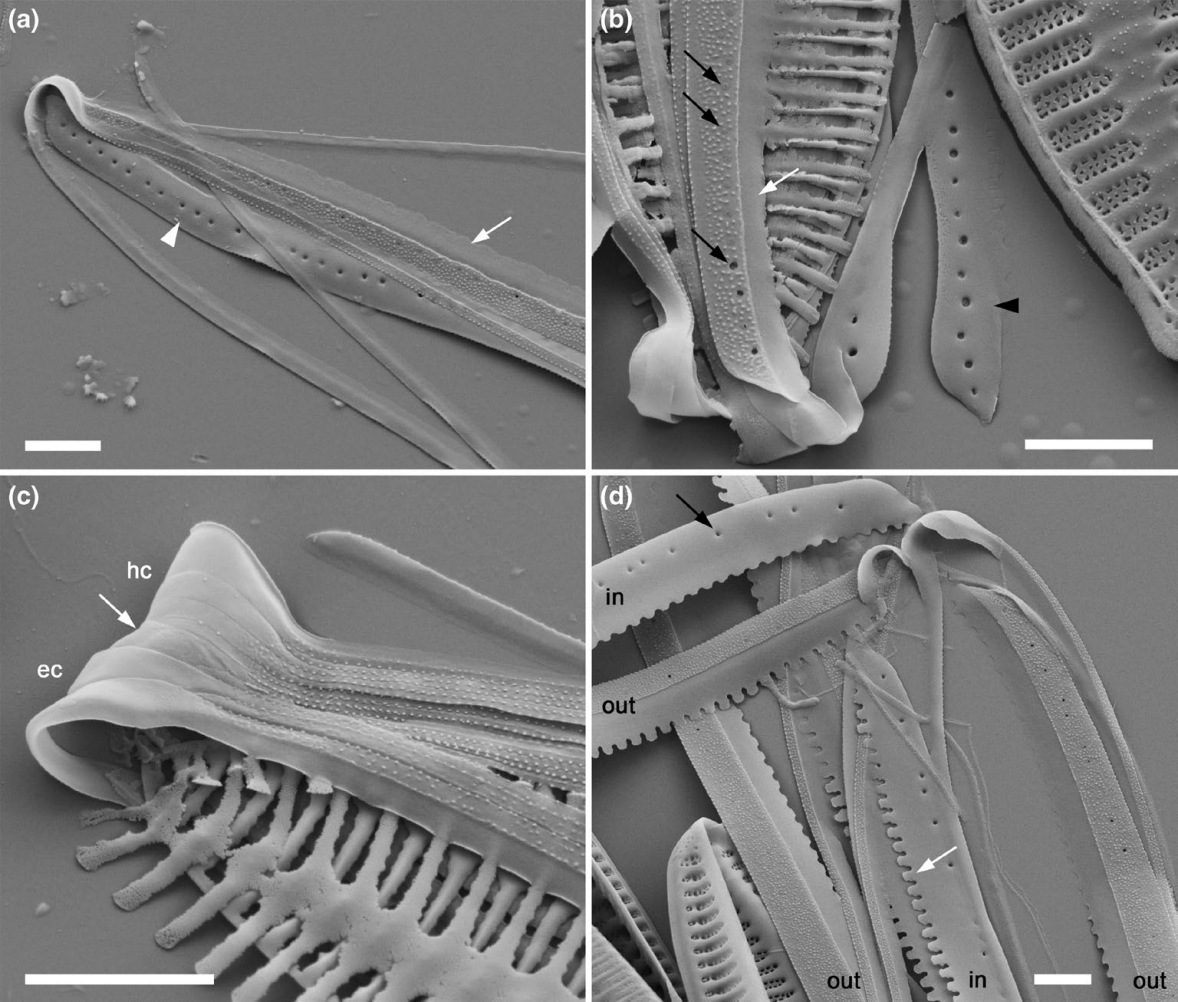
*Tryblionella* spp. girdle bands, (a, c) SEM *Tryblionella apiculata*, (d) *T. hungarica*. (a) Band 1 (valvocopula) and two more abvalvar bands. Band 1 bears warts externally on the pars exterior, while the internal surface (arrowhead) is smooth. Note that the pars interior of the valvocopula (white arrow) has an entire margin, also the line of round poroids (cf. (b)). (b) Disrupted frustule with band 1, which bears a fairly regular line of round poroids (e.g., black arrows) near the junction of pars exterior and pars interior; the pars interior has an entire margin (white arrow). The band interior is smooth (arrowhead). (c) Part of the girdle of a frustule from which the valves and valvocopulae have been detached, leaving only the abvalvar bands of the epicingulum (**ec**) and hypocingulum (**hc**) (the junction between these is marked by an arrow). All the bands bear small warts on the pars exterior and all are open. (d) *T. hungarica*. Detached bands from several thecae, whose valvocopulae are seen variously from the inside (**in**) or outside (**out**). Note the scalloped edges of the valvocopulae (e.g., white arrow) and the row of rather sparse poroids (e.g., black arrow): contrast (b). Scale bars = 2 μm.

Cingulum bands are open, with their open ends at alternate ends of the frustule, as in *Tryblionella apiculata* (Figure S5a). The valvocopula bears a rather irregular line of small poroids, which are closed by hymenes at their external apertures (Figure 6b). Externally, the pars exterior of the bands bears small warts and a smooth margin, while the pars interior has scalloped edges corresponding to the spacing of the valve striae (Figure 6d). Internally, the bands are entirely smooth (Figure 6d).

#### Tryblionella apiculata

**FIGURE 7:**
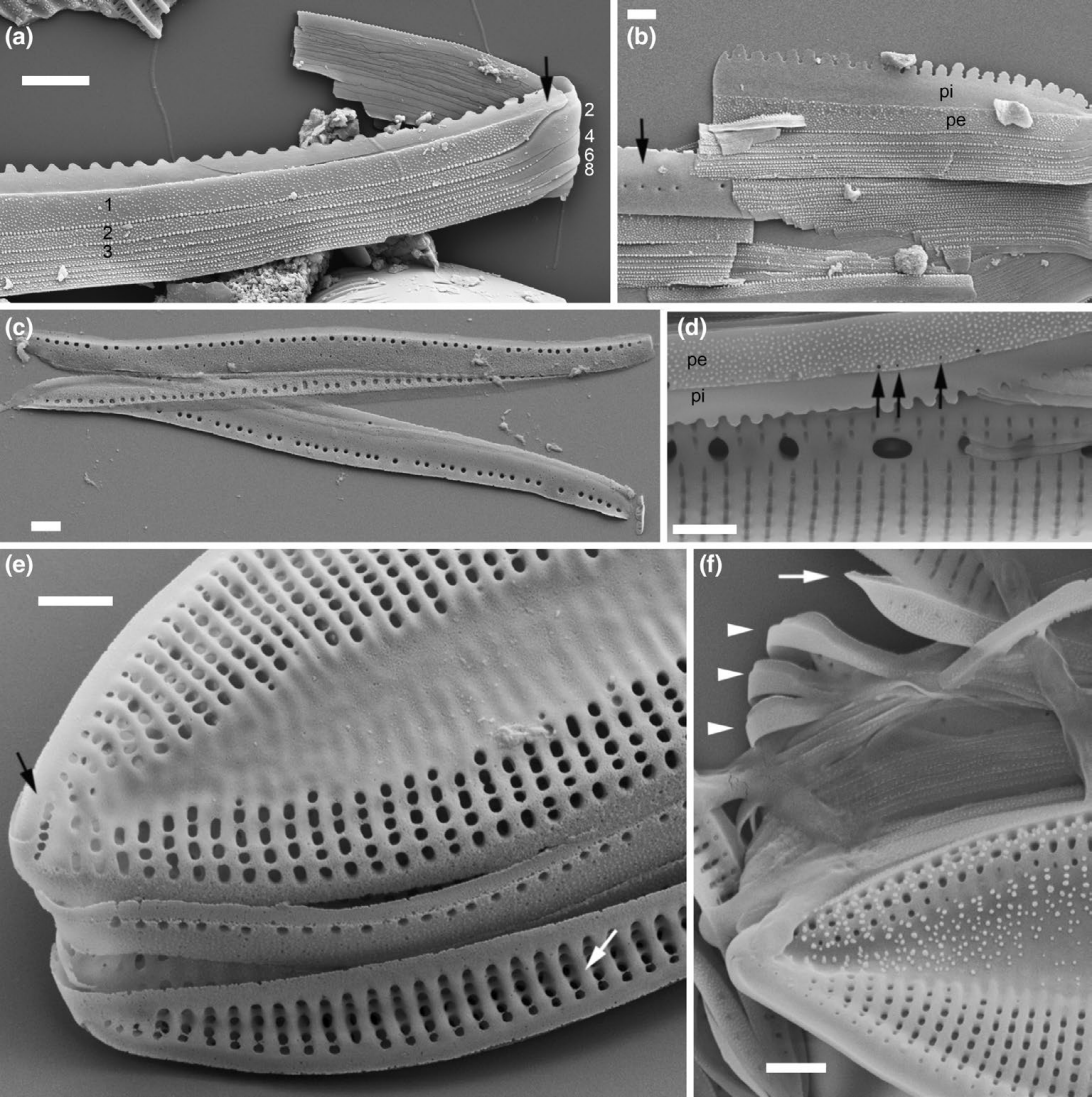
*Tryblionella* spp. girdle bands, (a–b) *Tryblionella apiculata*, (c, e) *T. gaoana*, (d, f) *T*. cf. *gaona*. (a) Detached cingulum with the open ends of bands 1 (arrow), 3, 5, 7, and 9, and the closed ends of bands 2, 4, 6, and 8 (numbered). The abvalvar margin of each band is marked externally by an orderly row of papillae. (b) Fragments of the girdle. Externally, all bands bear small papillae (see the pars exterior of band 1, **pe**) and band 1 bears a single row of small areolae. The pars interior (**pi**) and the internal face of band 1 (arrow) are smooth. (c) Detached fragments of bands: All are of band 1 (valvocopula), each with a single row of round areolae; the upper two fragments are lying with the exterior uppermost, and here the pars exterior appears rough, though without discrete papillae. (d) Center inside, overlain by part of band 1 (valvocopula). The fibulae are wide solid structures, separated by small rounded portulae; the central portula is wider. The smooth pars interior of the valvocopula (**pi**) bears short projections corresponding to the valve transapical ribs; the pars exterior (**pe**) bears densely spaced papillae and a single, irregular row of poroids (e.g., arrows). (e) Frustule pole. Note the absence of a marginal ridge and the presence of a row of special (non‐stria) areolae adjacent to the terminal fissure (black arrow); short striae of three to five areolae are present on the proximal mantle (e.g., white arrow). (f) Detail of disassembled frustule, showing the valve apex and multiple bands in the cingulum. The open end of band 1 and the closed ends of bands 2, 4, and 6 (arrowheads) are visible at this pole. Scale bars = 2 μm.

All the girdle bands bear small warts on the pars exterior like those present on the valve mantles, while the pars interior and the internal face of the pars exterior are smooth (Figures 6a,c and 7a,b). The first band (valvocopula) bears a single row of poroids on the pars exterior (Figures 6a,b and 7b), corresponding to the dots seen in LM. The bands are open, their orientations alternating so that the closed end of one band fills the gap left by the open ends of the adjacent band (Figures 6c and 7a). The bands are all very similar in structure, differing only in width and forming a graded series outward from the valve (Figure 7a).

#### Tryblionella gaoana

The first girdle band is broad (Figure 7e), with a fairly regular row of round poroids at the junction between pars exterior and pars interior (Figure 7c). The interior surface is entirely smooth, while it appears that the pars exterior bears delicate warts, though the specimens observed seem somewhat eroded (Figure 7c).

#### *Tryblionella* cf. *gaoana* Tokiane4 nitzG1 strain

The girdle bands are open, alternating in orientation (Figure 7f); there are at least six in the epicingulum. Externally, the pars exterior bears warts, and the first band (valvocopula) has a single irregular line of round poroids a little away from the junction with the pars interior, which is smooth (Figure 7d,f); the first band has a scalloped edge on the side that fits beneath the distal mantel of the valve (Figure 7d).

#### Tryblionella marginulata

**FIGURE 8:**
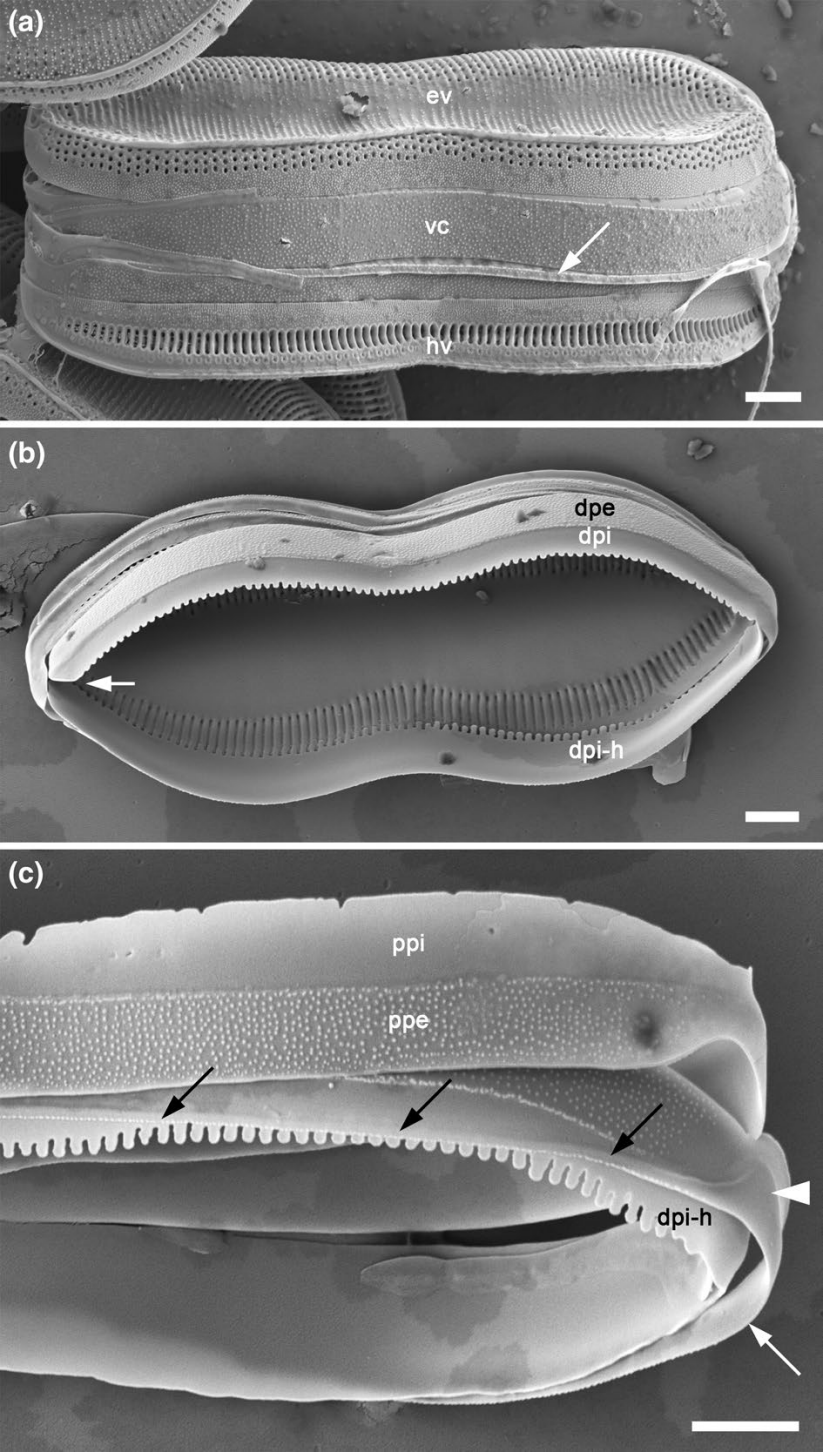
*Tryblionella marginulata*, girdle bands. (a) Frustule, girdle view, showing epivalve (**ev**) with a broad first band (valvocopula, **vc**) and a much narrower band 2 (arrow). (b) A frustule that has lost its epivalve, revealing the interior of the hypovalve and the structure of band 1 of the epicingulum, which is open (arrow) and has a wide pars exterior (**dpe**) and a smooth but crenulate pars interior (**dpi**) on its distal side. The valvocopula visible at the bottom (**dpi‐h**) is not part of the epitheca but belongs to the hypotheca (shown by careful observation of the right end of the theca). (c) Disrupted girdle, showing the proximal side of band 1, with rough pars exterior (**ppe**) and a smooth pars interior with a non‐crenulate margin (**ppi**). Below is the distal side of band 1 of the hypotheca (**dpi‐h**), characterized by its crenulate margin, overlain by the much narrower band 2 (arrows), which bears a large ligula (arrowhead) closing the gap left by the open ends of band 1 (cf. (b)). Scale bars = 2 μm.

The valvocopula (first band) is broad, while the rest of the cingulum bands are narrow (Figure 8). The interior surface of the bands is entirely smooth, whereas the exterior of each pars exterior bears small warts (Figure 8). The distal side of the valvocopula has a crenulate pars interior, which corresponds to the distal interior ribs of the valve (Figure 8b,c); the proximal margin is more or less smooth (Figure 8c, top).

### Morphological analysis

**FIGURE 9:**
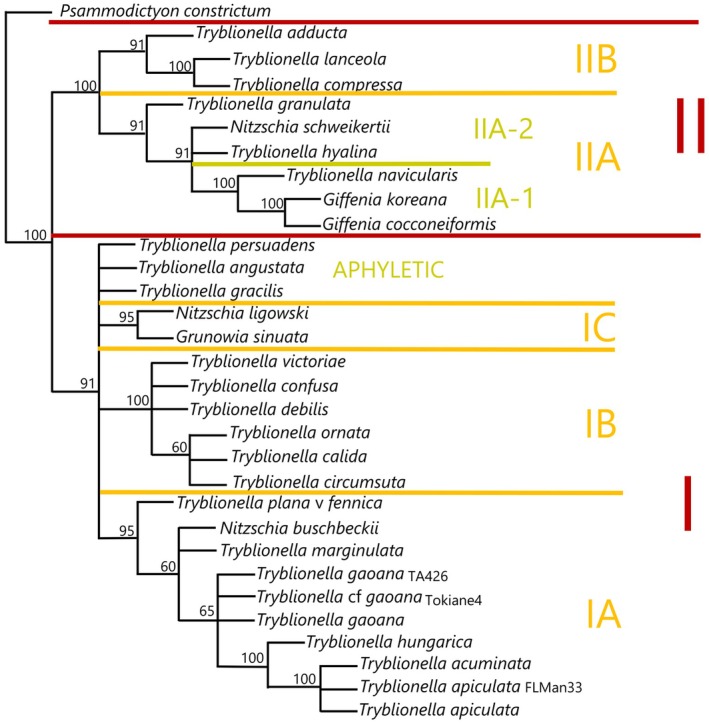
Majority rule consensus tree of *Tryblionella* and *Tryblionella*‐like taxa with available SEM data, based on morphological characters of valves listed in Table 1. Specific clades discussed in the text are labeled at the right of the tree. Numbers at nodes represent the percentage of which the clade at that node appears in the 23 most parsimonious trees.

The data matrix containing the character states of 31 *Tryblionella* s.l. taxa, for example, species currently described in *Tryblionella*, *Nitzschia*, *Giffenia*, and *Psammodictyon constrictum* as the outgroup, were used to perform the phylogenetic analysis based on 12 selected morphological characters. Based on the majority rule tree (Figure 9), an analysis of the synapomorphies (Table 2) revealed two major clades. In Clade I, we observed polytomy with three subclades, IA, IB, and IC, and three aphyletic taxa: *T. persuadens*, *T. angustata*, and *T. gracilis*. The Apiculatae taxa, for example, *T. apiuculata*, *T. acuminata*, *T. hungarica*, *T. gaoana*, *T. marginulata*, *T. plana* var. *fennica*, and *N. buschbeckii* constituted Subclade IA. *Tryblionella victoriae*, *T. confusa*, *T. debilis*, *T. ornata*, *T. calida*, and *T. circumsuta* represent Subclade IB, and *N. ligowskii* and *Grunowia sinuata* represent Subclade IC. The second Clade II consisted of two Subclades, A and B. Subclade IIA was split into two subclades: IIA‐1 included *T*. *navicularis* and *Giffenia* spp., and subclade IIA‐2 included *T. granulata*, *T. hyaline*, and *N. schweikertii*. Subclade IIB was represented by *T. adducta*, *T. lanceola*, and *T. compressa*. The individual characters were plotted on separate cladograms to help in proposing synapomorphic characters for each group (Figure S11 and Tables 1 and 2).

**TABLE 2:** List of character states of individual taxa (description of characters and their state is included in Table 1).

Taxon	Character											
	1	2	3	4	5	6	7	8	9	10	11	12
*Giffenia cocconeiformis*	2	2	1	0	1	0	–	–	0	0	0	0
*G. koreana*	2	2	1	0	1	0	–	–	0	0	0	0
*Grunowia sinuata*	0	2	0	3	1	0	1	0	–	0	1	0
*N. buschbeckii*	0	2	2	1	0	1	1	0	–	0	1	1
*N. circumsuta*	0	1	1	3	0	0	1	1	3	1	0	–
*N. lanceola*	0	1	0	1	0	1	0	0	–	0	0	2
*N. ligowski*	0	2	1	0	0	0	1	0	2	0	1	0
*N. schweikertii*	0	0	1	0	0	1	1	0	–	0	0	0
*Psammodictyon constrictum*	1	1	0	0	1	1	0	0	3	2	1	0
*Tryblioenlla acuminata*	0	2	2	2	1	1	0	0	1	0	1	–
*T. adducta*	0	1	0	1	0	1	0	0	–	0	0	0
*T. angustata*	0	1	0	3	1	1	–	0	2	0	1	1
*T. apiculata* FLMan33	0	2	2	2	1	1	0	0	1	0	1	2
*T. apiculata* TRY94CAT	0	2	2	2	1	1	0	0	1	0	1	2
*T. calida*	0	–	0	3	1	0	1	1	–	1	1	–
*T. compressa*	0	1	0	1	0	1	0	0	0	2	0	2
*T. confusa*	0	–	1	3	0	1	–	1	–	1	1	2
*T. debilis*	0	1	1	3	0	1	1	1	3	1	1	2
*T. gaoana* SZCZ CH97	0	2	2	0	0	1	1	0	1	0	1	2
*T. gaoana* TA426	0	2	2	0	0	1	1	0	1	0	1	2
*T.* cf. *gaoana* Tokiane4	0	2	2	0	0	1	1	0	1	0	1	2
*T. gracilis*	0	1	0	3	0	1	1	0	2	1	1	0
*T. granulata*	0	0	0	0	0	0	0	0	–	0	0	2
*T. hungarica* TRY981CAT	0	2	2	2	1	1	1	0	1	0	1	2
*T. hyalina*	0	0	1	–	0	–	0	0	–	0	–	0
*T. marginulata* PDK strains	0	2	2	2	0	1	1	0	1	1	1	1
*T. navicularis*	2	2	2	0	1	1	0	–	0	0	0	0
*T. ornata*	0	1	0	3	0	0	–	–	3	1	–	2
*T. persuadens*	0	1	0	3	0	0	1	0	1	0	0	1
*T. plana* var. *fennica*	0	2	2	3	0	1	1	0	1	1	1	0
*T. victoriae*	0	1	0	1	0	1	1	1	–	1	–	–

Abbreviation: –, missing data.

### The Apiculatae taxa characteristics

Apiculatae taxa (Figure 9, Clade IA) can be distinguished and characterized by three key features: (1) a porose valve cross‐section (Figure 1b); (2) longitudinal valve undulation, where the peak of the undulation is located on the proximal side of the valve (Figure 1d); and (3) the presence of an axial sternum with the visible thickened virgae bisecting the sternum, completely or incompletely (Figures 3b and S11). Other morphological characteristics analyzed exhibited variability across the species in this clade. Some characters have also been observed in taxa outside of Apiculatae, as outlined in the subsequent discussion. The marginal ridge was present in various forms across nearly all species (Figure 3b,c), with the exception of a secondary loss in *Tryblionella gaoana*. The majority of species of Apiculatae possessed uniseriate striae (Figure 3b), except for *T. hungarica*, *T. apiculata*, and *T. acuminata* (Figure 2c). All Apiculatae had unevenly thick virgae (Figure 2c), although this characteristic was plesiomorphic. The virga‐to‐fibulae ratio was described as a binary character—either 1:1 or greater than 1:1—to accommodate the earlier classification of *Tryblionella* on the basis of “indistinct” fibulae, which likely were occluded by the virgae. Under this scheme, only *T. acuminata* and *T. apiculata* in Clade I presented a 1:1 virga‐to‐fibulae ratio (Figure 2b). The remaining species had higher ratios, but there was variability within that group regarding the number of virgae compared to fibulae. For instance, *T. marginulata* had a ratio of 3:1 (Figure S6), while *T. plana* var. *fennica* had an irregular ratio (Figure S9e). Furthermore, the fibulae in Apiculatae taxa were solid and lacked any poroids or ornamentation (Figure 2a). All Apiculatae taxa shared similar proximal raphe endings, straight and nestled in a notch. The morphology of the ornamentation on the proximal exterior valve varied among taxa. In most Apiculatae species, areolae showed no change in morphology or arrangement toward the raphe canal; however, in *T. plana* var. *fennica* and *T. marginulata*, areolae changed from porose to linear morphology (Figure 1b). In contrast, all analyzed representatives of the Apiculatae group demonstrate the presence of pores on the outer side of the raphe canal (Figures 1b, 2c and S11, Table 2).

### Non‐Apiculatae taxa

A synapomorphic character for taxa within Clade IB (Figure 9) is the presence of perforated fibulae (Figure 2f), which were not observed in the other analyzed taxa. Major taxa from Clade IB (noting the missing data for *Tryblionella confusa* and *T. calida*) demonstrated longitudinal valve undulation. However, unlike Apiculatae taxa, the undulation in representatives of Clade IB peaked on the distal side of the valve (Figure 1a). Two species from Subclade IC belonged to different genera and possessed distinguishing characteristics that set them apart from the Apiculatae taxa: *Grunowia sinuata* lacked an axial sternum, while *Nitzschia ligowskii* had an axial sternum without visible thickened virgae. Aphyletic taxa, including *T. persuadens*, *T. angustata*, and *T. gracilis*, also displayed longitudinal valve undulation. However, similar to taxa from Subclade IB, the peak of the undulation in these species was located on the distal side of the valve. Additionally, in these taxa, the axial sternum was either absent or indistinct. Taxa from Clade II showed a significant difference in that they lacked areolae on the raphe canal, in contrast to taxa from Clade I, with the exceptions being *T. persuadens* and *T. circumsuta*. The taxa within Subclade IIA‐1 showed a distinct alveolate valve cross‐section (Figure 3c), while taxa in Subclade IIA‐2 lacked any noticeable longitudinal valve undulation. Taxa from Subclade IIB demonstrated the undulation with the peak located on the proximal side of the valve (Table 2).

**FIGURE 10:**
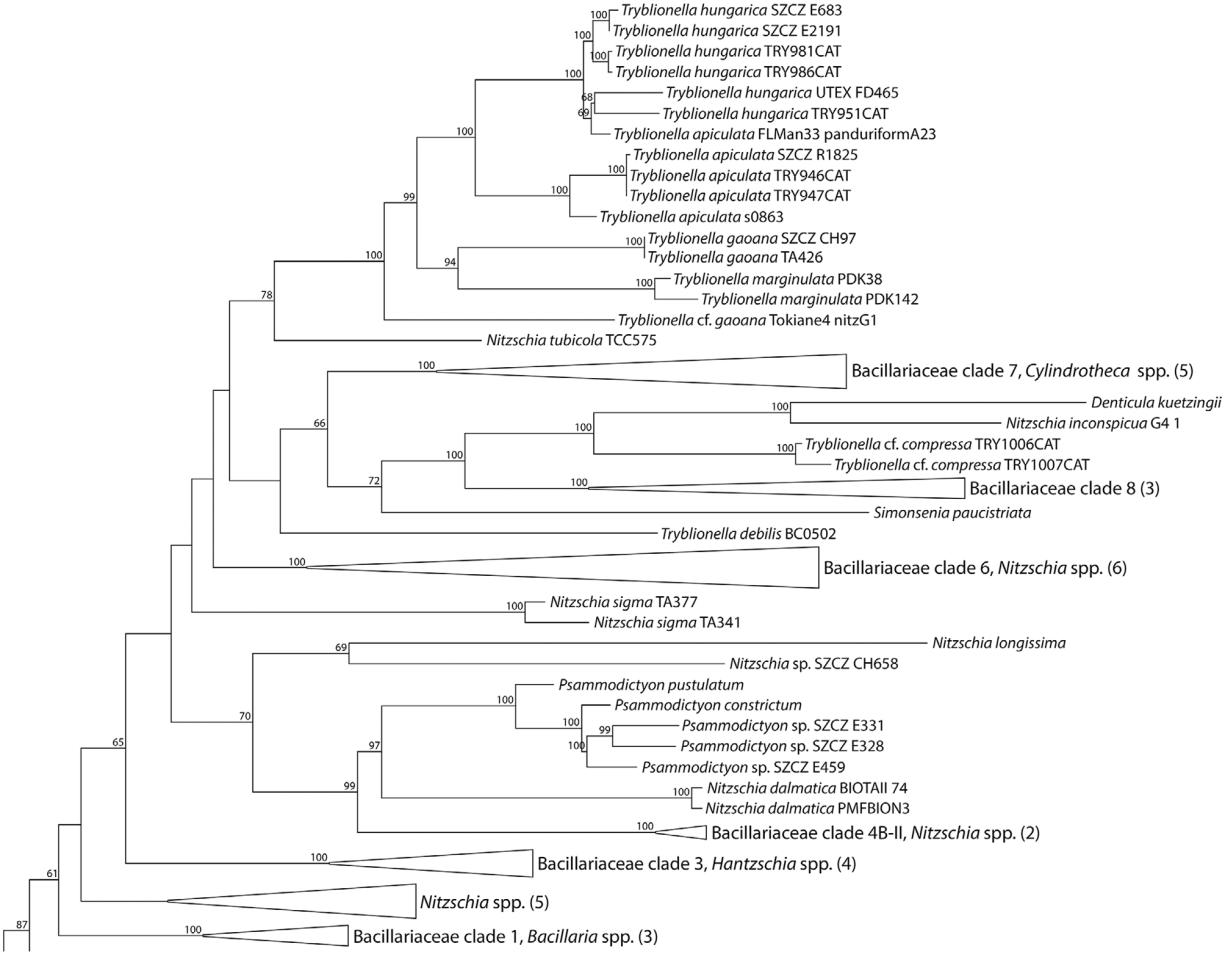
A clade of Bacillariaceae cut from a Maximum Likelihood tree inferred from the alignment of concatenated *rbcL*, *psbC*, and SSU genes of 151 diatom taxa rooted with *Triparma pacifica*. Bootstrap values below 50 were omitted.

### Molecular analysis

To investigate the phylogenetic relation between *Tryblionella* spp. and other Bacillariaceae, we performed maximum likelihood phylogenetic analysis based on DNA sequences downloaded from GenBank, in addition to those obtained in this study. A three‐gene dataset (*rbc*L, *psb*C, and SSU) from 151 diatom taxa was used, and the tree was rooted with a strain of *Triparma pacifica*.

The topologies displayed by the three‐gene inferred tree (Figure 10) featured a monophyletic Bacillariales. *Tryblionella hungarica* isolates were in a clade sister to a *T. apiculata* clade with high bootstrap support (100); however, neither species was monophyletic due to the presence of *T. apiculata* strain FLMan33 panduriformA23 inside the *T. hungarica* clade with 63% support. Together, these two clades were sister (99% bootstrap support) to a clade comprising *T. gaoana* (TA426 and SZCZ CH97) and *T. marginulata* (RebReef3_elongpeanutPDK142, RebReef2_pandurifPDK38), which had somewhat lower bootstrap support (94%). Beyond all these was *Tryblionella* cf. *gaoana* (isolate Tokiane4 nitzG1). The whole *Tryblionella* clade, including all the above, received 100% support. This was nested in a larger clade with other genera of Bacillariaceae, namely *Nitzschia*, *Cylindrotheca*, *Denticula*, *Simonsenia*, *Hantzschia*, *Psammodictyon*, and *Bacillaria*. Relationships among these were not well resolved. Two other “*Tryblionella*” species were included in our analysis: *T. compressa* (two isolates) and *T. debilis*. None of these grouped with *T. hungarica* and *T. apiculata* (Figure 10). Due to discrepancies between the molecular phylogenetic analyses and the morphology‐based identification concerning the position of *T. apiculata* strain FLMan33 panduriformA23, in which both phylogenies placed in the *T. hungarica* clade (Figure 10), an approximately unbiased test (AU) test was performed, comparing the “best trees” presented in Figure 10 to a tree with a constrained topology, in which strain FLMan33 panduriformA23 was put in the *T. apiculata* clade. The test showed that the likelihood scores of both the constrained and “best” tree were not significantly different, which suggests that the placement of strain FLMan33 panduriformA23 should be viewed as ambiguous. The results are at https://doi.org/10.5281/zenodo.10997217.

## DISCUSSION

### Phylogenetic and taxonomic position

The status of *Tryblionella* as a distinct genus from other taxa in the Bacillariales has been questioned for many years due to the difficulty in finding clear diagnostic characters separating it from *Nitzschia* (Cleve & Grunow, 1880; Hustedt, 1930; Krammer & Lange‐Bertalot, 1997). The development of high‐quality SEM technology seemed to provide evidence for the status of *Tryblionella* as a separate genus (Round et al., 1990), showing features in many *Tryblionella* species that are rare in other Bacillariales, such as the axial sternum and marginal ridge, adding to the overall Gestalt of the genus visible in LM: broad valves, highly eccentric but inconspicuous raphe system and fibulae, and frequently undulate valve face. However, diagnosis of the genus was still difficult, and Round et al. (1990) offered four alternative character combinations to define it. Molecular phylogenies (Mann et al., 2021; this paper) have shown, however, that one reason for this difficulty was that the 1990 idea of *Tryblionella* was not monophyletic, contradicting Round et al.'s confident assertion that it was “a natural group.” Our phylogenetic analyses, based on morphological characters with support of a three‐gene concatenated dataset (*rbc*L, *psb*C, and SSU), defined synapomorphic features and extended the earlier one‐ to multigene trees of Mann et al. (2021) with new accessions and taxa. All showed that there are several clades and subclades of *Tryblionella* taxa.

The taxonomic consequences of these data cannot be decided without reference to broader problems in the Bacillariaceae, caused by the fact (now well established, e.g., Mucko et al., 2021; Rimet et al., 2011; Mann et al., 2021) that the largest genus, *Nitzschia*, is paraphyletic with respect to several smaller, well‐defined monophyletic genera, including *Pseudo‐nitzschia*, *Fragilariopsis*, *Denticula*, *Cylindrothecai*, and *Simonsenia*. One solution to this would be to subsume all of these into *Nitzschia*, which, indeed, is where some of them started. However, given how important and familiar some of these genera are in ecological, toxicological, and genetic studies, we support a strategy presented by Mann et al. (2021) in which *Nitzschia* is split into monophyletic groups, preferably groups that have a clear morphological signature so that they can be identified in LM; a few of the many possible options have been outlined by Mann et al. (2021). From this perspective, the Apiculatae clade is a strong candidate for generic status.

A key issue is, therefore, whether the Apiculatae clade can be called *Tryblionella*, and this, in turn, depends on the relationships of *T. acuminata*—the type of *Tryblionella*. There are no molecular data for this species, but the valve structure of *T*. *acuminata* has been documented by Mann (1978; Figure S10) and Guttinger (1989). *T. acuminata*, as a typus generis, has all three characters of the Apiculatae group defined here, suggesting the monophyletic nature of this group and its suitability to be called *Tryblionella*. All these characteristics can be observed by careful examination in LM, which makes it easier to recognize characters of *Tryblionella* s.s. also appears to share a similar girdle structure based on available data: The girdle is graded (i.e., all the bands have a similar structure but gradually change in width from the valve outward), except that the first band (valvocopula) bears a single row of poroids in the pars exterior and a crenulate margin on the side corresponding to the distal valve mantle. Most species also have a thin continuous marginal ridge at the junction of the valve face and distal mantle, and most have scattered small warts (papillae), or a dense covering of them, on part or all of the valve face, the mantles, and the girdle bands.

Our conclusion is, therefore, that Round et al.'s (1990) decision to resurrect the genus *Tryblionella* is consistent with available phylogenetic and morphological data, but they were wrong to extend *Tryblionella* beyond Grunow's Apiculatae group to include *T. debilis* (and by extension *T. gracilis*, *T. levidensis*, and similar species) or *T. compressa*. The results of the conducted research indicate, however, that *Nitzschia buschbeckii* has all the characteristics of *Tryblionella* s.s. and, therefore, should be recognized as *Tryblionella*:

***Tryblionella buschbeckii*** (Witkowski, Lange‐Bertalot & Ruppel) R.M. Olszyński, D.G. Mann & M. Ashworth comb. nov.

Basionym: *Nitzschia buschbeckii* Witkowski, Lange‐Bertalot & Ruppel (2004), p. 583, figures 10–25, in Witkowski, A., et al. Four new species of *Nitzschia* sect. Tryblionella (Bacillariophyceae) resembling *N. parvula*. *Phycologia, 43*(5), 579–95. https://doi.org/10.2216/i0031‐8884‐43‐5‐579.1.

Diagnosis: *Tryblionella buschbeckii* has characteristics of *Tryblionella* sensu stricto, for example, porose cross‐section and longitudinal valve undulation with a peak on the proximal side of the valve and axial sternum completely or incompletely interrupted with thickened virgae (Witkowski et al., 2004).

### Relationships within *Tryblionella*

Both phylogenetic analyses based on morphology and molecular markers support a clade within *Tryblionella* (s.s.) containing *T. apiculata*, *T. hungarica*, and *T. acuminata* (the last only from morphological analysis). That group of taxa differs from the rest of Apiculatae by the bi‐or multiseriate striae. One key difference between *T. apiculata* and *T*. *hungarica* has always been the virga‐to‐fibula ratio, which is greater than 1:1 in the latter species. However, some strains had unclear phylogenetic positions or conflicting morphology. The molecular analysis indicated that strain FLMan33 panduriformA23 might be more closely related to *T. hungarica* than *T. apiculata* despite having fibulae on almost all transapical ribs. Nevertheless, its position relative to strains FD465 and TRY951CAT had very low bootstrap support. Whereas strain FD465 was originally identified as *T. apiculata*, LM photos published on ProtistCentral https://www.protistcentral.org/Photo/get/photo_id/1203 show a relationship between the fibulae and striae that actually corresponds to *T. hungarica* (Theriot et al., 2010). Strain TRY951CAT was originally identified as *T. hungarica*, which can be confirmed based on SEM photos online https://websites.rbge.org.uk/algae/research/Bacillariaceae_images/TRY951CAT.pdf (Mann et al., 2021). Both strains, FD465 and FLMan33 panduriformA23, were isolated from cells collected in distinct environments, including tropical regions and the skin of manatees inhabiting a warm river (Frankovich et al., 2018; Theriot et al., 2010). The variations in ecological origins may “explain” the morphological differences observed between these strains and strains of *T. apiculata* from Europe. (That there is a further level of taxonomic differentiation that cannot yet be clarified sufficiently to recognize any taxa formally.) Based on our morphological analysis and low bootstrap support of molecular analysis, we identified strain FLMan33 panduriformA23 as a *T. apiculata*. According to the phylogenetic analysis based on molecular markers, strain *Tryblionella* sp. s0863 (Mann et al., 2021) is closely related to the *T. apiculata* clade, which is confirmed by the morphology, including the relation between the virga and fibulae.

Isolate TA426 was originally identified as *Nitzschia ligowskii* by An et al. (2017). This species and *Tryblionella gaoana* are morphologically similar (Witkowski et al., 2004, 2016) and difficult to distinguish based solely on LM observations (as done by An et al., 2017, who likely wrote their paper before *T. gaoana* was described). Witkowski et al. (2016) emphasize differences in the structure of the proximal mantle: “only a single row of relatively large areolae occurs on the proximal mantle of *N. ligowskii*, whereas three rows of areolae occur in *T. gaoana*” (p. 191). Thanks to An et al.'s generosity, we were able to determine that TA426 has the *gaoana* structure (Figure 7e; Witkowski et al., 2004, 2016) The topology of the three‐gene tree (Figure 10) places strain TA426 next to the *T. gaoana* type strain (SZCZ CH97), with maximum bootstrap support. Hence, with the support of data from morphological observations, this strain is re‐identified as *T. gaoana*.

Strain Tokiane4 nitzG1 is a paradox. It shares all the analyzed morphological characteristics with *Tryblionell. gaoana*, although it has a slightly denser striation and wider fibulae than *T. gaoana* and only a few scattered pores on the valvocopula pars exterior, unlike the clearly visible and dense pores in *T. gaoana*. However, the molecular phylogenetic analysis does not support a sister relationship between this strain and *T. gaoana*. Instead, it branches off at the base of the *Tryblionella* clade. Further taxon sampling in *Tryblionella* is pending, and we have left the species undetermined for now.

*Tryblionella marginulata* was originally described by Grunow as *Nitzschia marginulata* (in Cleve & Grunow, 1880) and transferred to *Tryblionella* by Round et al. (1990). Several varieties and forms were also described by Grunow that were not illustrated at the time (Cleve & Grunow, 1880); further varieties and forms have since been described (see Simonsen, 1987; Van Landingham, 1978), but few are included in identification texts, nor are they often reported (Krammer & Lange‐Bertalot, 1997; Witkowski et al., 2002). The lack of drawings or photographs and full documentation causes difficulties with identification. However, Louvrou and Economou‐Amilli (2016) proposed two forms of *T. marginulata*—*elongata* and *parva*—whose valve sizes correspond to the bigger and smaller forms of the strains we studied. The three‐gene analysis revealed a close relationship between two *marginulata* strains, RebReef3_elongpeanutPDK142 and RebReef2_pandurifPDK38, with maximum support. Analysis of the literature (Louvrou & Economou‐Amilli, 2016; Witkowski et al., 2002) confirms that these two forms can co‐occur together in one environment; elsewhere and for the moment, we treat both sequenced strains as an undifferentiated *T. marginulata*.

### Ontogeny

It has been suggested that valve ontogeny can help in the interpretation of morphology and systematics in diatoms (Cox, 2012; Mann, 1984), and the examination of *Tryblionella* species supports this idea. Ontogeny indicated, first, that the axial and marginal sterna of *Tryblionella* species (in all three species, *T. apiculata, T. hungarica*, and *T. debilis*) are not pattern centers. In other words, they are not primary elements of the valve, formed in the earliest stages of silicification, and the pattern is not formed outward from them in the same way as occurs from the annulus of centric diatoms (Sato, 2010), the axial sternum of araphid pennates (Sato et al., 2011) or the raphe sternum of both monoraphid and biraphid diatoms (Chiappino & Volcani, 1977). The axial and marginal sterna of the *Tryblionella* species are thus secondary structures formed by fusion after the transapical rib system is already substantially complete. However, *T. hungarica* and *T. apiculata* differ from *T. debilis* in terms of when and how the secondary sterna are initiated. In *T. hungarica* and *T. apiculata*, the sterna are formed by localized fusion between adjacent transapical ribs before the areolae are delimited; in *T. debilis*, conversely, the areolae are formed before the sterna, which are therefore created by infilling of existing areolae. This difference correlates with the different phylogenetic positions of *T. apiculata* and *T. hungarica*, which represent clade 5B of Mann et al. (2021), whereas *T. debilis* is in clade 5A and its closest known relative is a species, *Nitzschia tubicola*, that entirely lacks sterna or any other obvious characteristic of *Tryblionella*‐like diatoms. Our hypothesis is, therefore, that the sterna and folding of the valve in *T. debilis* have evolved independently of those in *T. hungarica* and *T. apiculata*. To test this idea further will require (1) better genetic sampling and ontogenetic studies of those *Tryblionella* species likely to be related to *T. debilis*, judging by their similarly complex fibulae (e.g., *T. levidensis*, *T. gracilis*); and (2) ontogenetic studies of *T. gaoana* and *T. marginulata*, where, although sterna are present, the striae are uniseriate, containing simple round poroids as in *T. debilis*. Our observations suggest a potential relationship between the presence of thickened, relief virgae within the axial sternum, and the development of the primary axial sternum. However, the limited sampling in our ontogenetic analysis, confined to three taxa, constrains our ability to definitively establish this relationship.

### Implications for the taxonomic investigation of other “*Tryblionella*” species

Our phylogenetic analysis based on selected characteristics suggests a direction for further studies on *Tryblionella* s.l. taxa. Clade I includes several taxonomical problems, one of which is Apiculatae, which was studied in this paper. Although *T. debilis* can be found in Clade I (Figure 9), it was resolved in a clade distinct from the Apiculatae group, in which the common features are a peak in the valve undulation on the distal side of the valve and porous fibulae (see also Mann et al., 2021). The remaining taxa from Clade I, including *Nitzschia ligowski, Grunowia sinuata, T. persuadens, T. angustata*, and *T. gracilis*, require a new morphological and molecular analysis supported by a broader taxon sampling campaign, perhaps targeted at taxa with sail‐like marginal ridges, areolae on the raphe canal tube, and a fibula:virga ratio greater than 1:1. In Clade II (Figure 9), we have distinguished several subclades that may be subject to deeper analysis. The cross‐section of the alveolate valve, characteristic of *Giffenia* spp. (Round & Basson, 1997) within *Tryblionella* s.l., strongly supports a re‐investigation of *T. navicularis*, which seems to share this same valve structure. The remaining species within Clade II exhibit porose valve cross‐sections; however, species from Subclade IIA‐2—specifically *N. schweikertii*, *T. hyalina*, and *T. granulata*—are the only taxa analyzed that lack longitudinal valve undulation. Molecular analyses concerning the taxonomical position of *T. compressa* (Mann et al., 2021; Mann & Trobajo, in press), which serves as a representative of Clade IIB, along with Pantocsek's (1902) classification of this species into a separate genus, *Zotheca* (as *Z. punctata*), support our findings about its distant relationship from *Tryblionella* s.s.

Our research focused specifically on the Apiculatae group, within which the species *Tryblionella typus generis* is categorized, with the aim of identifying diagnostic characters pertinent to the genus *Tryblionella*, but it now needs to be extended to other *Tryblionella*‐like Bacillariaceae; *Tryblionella*‐like Bacillariaceae remain inadequately studied in terms of morphology and are poorly represented in both molecular and morphological phylogenetic reconstructions. Culturing large‐celled diatoms poses significant challenges; nevertheless, culturing them is crucial for acquiring high‐quality morphological data and achieving improved resolution of molecular phylogeny, both of which are essential for more precise taxonomical classification. In addition, collecting SEM data for *Tryblionella* taxa held in collections is critical for expanding the breadth of morphological characters and taxonomic sampling in and around this genus. These diatoms are predominantly located in brackish or marine environments and are rarely encountered in considerable abundance.

## AUTHOR CONTRIBUTIONS

**Rafał M. Olszyński:** Conceptualization (lead); data curation (supporting); formal analysis (equal); funding acquisition (equal); investigation (equal); methodology (equal); project administration (lead); resources (equal); software (supporting); supervision (lead); validation (supporting); visualization (supporting); writing – original draft (lead); writing – review and editing (lead). **Ewa Górecka:** Conceptualization (supporting); data curation (equal); formal analysis (supporting); funding acquisition (equal); investigation (equal); methodology (equal); project administration (supporting); resources (equal); software (equal); supervision (supporting); validation (supporting); visualization (supporting); writing – original draft (supporting); writing – review and editing (equal). **Rosa Trobajo:** Conceptualization (equal); data curation (supporting); formal analysis (supporting); funding acquisition (equal); investigation (supporting); methodology (supporting); project administration (supporting); resources (equal); software (equal); supervision (supporting); validation (supporting); visualization (supporting); writing – original draft (supporting); writing – review and editing (equal). **Romain Gastineau:** Data curation (supporting); formal analysis (supporting); methodology (supporting); resources (supporting); software (supporting); visualization (supporting). **Matt Ashworth:** Conceptualization (equal); data curation (lead); formal analysis (equal); funding acquisition (equal); investigation (equal); methodology (equal); project administration (supporting); resources (lead); software (supporting); supervision (equal); validation (equal); visualization (supporting); writing – original draft (supporting); writing – review and editing (equal). **David G. Mann:** Conceptualization (lead); data curation (equal); formal analysis (lead); funding acquisition (equal); investigation (lead); methodology (lead); project administration (supporting); resources (lead); software (lead); supervision (lead); validation (lead); visualization (lead); writing – original draft (equal); writing – review and editing (lead).

## Supporting information


**Figure S1.** *Tryblionella apiculata*, clone TRY946CAT, SEM. (a) Whole valve, exterior. (b) Valve interior, central part, showing the axial sternum interrupting the striae and the fibulae, which have a 1:1 relationship with the transapical ribs, except at the center; here, the relationship is lost, and there is a wider interspace (**ci**). (c) External detail of center. Note the notch in the raphe canal containing the central raphe endings, the multiseriate striae containing irregularly shaped poroids, the thickened transapical ribs (virgae), and small warts on the axial sternum. The striae continue into the wall of the raphe canal (arrow). (d) Valve mantle with very short multiseriate striae; at least part of each stria (e.g., at arrow) opens into the raphe canal. (e) The valve end with the terminal raphe fissure hooked toward the narrow (proximal) side of the valve. Note the small warts on the (distal) mantle (arrow), the small marginal ridge separating the distal mantle from the valve face, and the row of apparently simple poroids on the mantle just beneath the marginal ridge. (f) The valve end with the terminal raphe fissure hooked toward the wider (distal) side of the valve. Scale bars = 2 μm.

**Figure S2.** *Tryblionella apiculata*, isolate FlMan33, (a–b) LM and (c–o) SEM. (a–b) Valves: raphe to the left: note the irregularities in the line of fibulae (e.g., arrowheads) and transapical striations that appear to traverse the whole valve face, except for short strips near the poles (arrows). (c) Calve exterior. (d) Frustule, highly tilted, showing a strongly undulate valve face and a marginal ridge (arrow) separating the valve face and distal mantle, and a single row of round areolae (e.g., arrows) along the mantle. (e) Center, with raised and keeled raphe and central raphe endings. (f) Valve pole, exterior, with biseriate striae interrupted by an axial sternum with a dense scatter of papillae. A plainer area is present near the poles (cf. arrows in (a–b)). (g) Oblique view of the proximal mantle with biseriate striae (e.g., arrow). (h) Proximal mantle with biseriate striae (black arrows) extending into the raphe canal wall (top arrow). Inside (below), the valve surface is smooth. Note that the distal striae (e.g., white arrow) are simple, with no sign of the extra mantle areolae present externally (cf. (d)). (i) Valve interior: fibulae generally spaced one per transapical rib, but sometimes getting out of phase (e.g., arrowhead). (j) Valve center inside with larger central portulae and an unusually large fibula (arrow). (k) Valve pole, inside. (l) Broken valve showing the raphe canal (arrow) and fibula structure. (m) Fragments of the girdle. Externally, all bands bear small papillae (see the pars exterior of band 1, **pe**) and band 1 bears a single row of small areolae. The pars interior (**pi**) and the internal face of band 1 (arrow) are smooth. (n) Detached cingulum with the open ends of bands 1 (arrow), 3, 5, 7, and 9, and the closed ends of bands 2, 4, 6 and 8 (numbered). The abvalvar margin of each band is marked externally by an orderly row of papillae. (o) Forming valve lying within the valve of the parent cell: transapical ribs beginning to fuse to form axial sternum. Scale bars = 2 μm.

**Figure S3.** *Tryblionella apiculata*, isolate s0863, LM (a, b, i, j) and SEM (c–h, k; all with 25° tilt). SEM indicates that the material was somewhat eroded, lacking occlusions in the areolae and with evidence of pitting in the silica basal layer. (a, b) Valves. (c) Valve exterior. (d) Theca, seen from the distal side. Note the presence of a marginal ridge separating the valve face from the distal mantle (**dm**), which contains short striae of triplet or doublet areolae (corresponding to the tri‐ or biseriate striae of the valve face). The first band (valvocopula, **vc**) is somewhat wider than the second (arrow). The mantle and girdle bands bear densely packed papillae. (e) Valve center outside. Note triseriate striae, which extend into the raphe canal and central raphe endings. (f) Valve interior: each transapical rib (virga) bears a fibula, except at the center (see g). (g) Valve center, inside. The center most portula is wider than the others. (h) Valve pole, outside, with distally curved terminal fissure and a distinctive row of poroids nearby (arrow) that do not relate to valve face striae. (i, j) Two examples of bands 1 and 2. Both bands are open. The first (valvocopula) bears a single row of areolae, while the second is much narrower and lacks areolae. (k) Stage in valve formation, in which the axial sternum is already complete, and the areolae on either side of the transapical ribs are almost completely delimited, but the third (central) row of areolae of the triseriate striae is only incipient. At this stage, the fibulae are still absent (the side of the valve corresponding to the proximal mantle has snapped off). Scale bars = 10 μm (a, b, i, j), 5 μm (c, d, f) or 1 μm (e, j, h, k).

**Figure S4.** *Tryblionella hungarica*, clone TRY981CAT, SEM. (a) Whole valve, interior. (b) Valve interior, central part, with wider central interspace (**ci**). (c) Valve interior, part showing the axial sternum interrupting the striae and the fibulae, which do not have an exact spatial relationship with the transapical ribs, there being many fewer fibulae. (d) Part of valve exterior, showing biseriate striae, axial sternum, thickened transapical ribs (virgae), small marginal ridge, a row of apparently simple poroids just beneath the marginal ridge, and small warts on the mantle. (e) Proximal mantle, showing lines of small warts extending to the valve margin (black arrow) and very short multiseriate striae, with at least part of each stria (e.g., white arrow) opening into the raphe canal. (f) External detail of center. The largely biseriate striae contain irregularly shaped poroids (often shaped like clover leaves) and continue into the wall of the raphe canal (e.g., at arrow), where the poroids become smaller and simpler. (g) The valve end with the terminal raphe fissure hooked toward the wider (distal) side of the valve. Note that the raphe opens onto the crest of a narrow ridge (see also (f)). Scale bars = 2 μm, except (a) (10 μm) and (e) (1 μm).

**Figure S5.** *Tryblionella hungarica* SEM. (a, d) Strain SZCZ E683 and (b, c) strain TRY981CAT. (a) Intact frustule in girdle view, showing the girdle composed of several narrow bands and a row of single round areolae on the distal mantle (arrow), which lie just below a slim marginal ridge; note also the contrasting structure of the distal and proximal mantles. (b) Valve interior, center, showing a stria that bridges the sternum (arrow). (c) Valve pole, exterior. Note the single round areolae on the distal mantle (e.g., black arrows) and a group of poroids near the terminal fissure of the raphe that resembles a pore field (white arrow). (d) Interior of valve pole, showing the group of closely spaced small pores in the “pore field” (arrow). Scale bars = 10 μm (a) or 1 μm (b–d).

**Figure S6.** *Tryblionella marginulata* (a–b) LM, (c–j) SEM. (b, f–h) Isolates PDK38 and (a, c–e, i–j) PDK142 LM. (a–b) Valves. (c) Whole valve, exterior, zero tilt. (d) Frustule tilted; note the prominent fold of the valve face. (e) Detail of the valve center in (c) with central raphe endings (arrow). (f–g) External and internal views of valves. (h) The valve pole tilted. Note the double row of areolae along the raphe canal (arrow), the scatter of small papillae on the valve face and mantle, and the presence of a marginal ridge, below which are two or three areolae (e.g., arrowhead) in each stria. (i) Oblique view of the interior of the proximal half of the valve, showing the fibulae and a single row of areolae (e.g., arrows) opening into the proximal wall of the raphe canal. (j) Fractured valve; note the complex structure of the fibulae. Scale bars = 10 μm (a–b), 5 μm (c–f), or 1 μm (e, h–j).

**Figure S7.** *Tryblionella* spp., SEM (25° tilt, unless stated otherwise). (a) *Tryblionella* sp. isolate Tokiane4 HK533, whole valve exterior, zero tilt, for comparison with (b) *T. gaoana*, isolate SZCZCH97 (see Witkowski et al. 2016), highly tilted: proximal mantle with short striae of 3 areolae (e.g., arrow). (c–h) *T. gaoana* isolate TA426. (c) Whole valve, exterior, zero tilt. (d) Valve interior. (e) Valve pole, with terminal fissure curving toward the distal side and raphe opening onto the crest of a narrow ridge. Two poroids in each stria open into the raphe canal (arrow). (f) Frustule pole. Note the absence of a marginal ridge and the presence of a row of special (non‐stria) areolae adjacent to the terminal fissure (black arrow); short striae of 3–5 areolae are present on the proximal mantle (e.g., white arrow). (g) Valve center outside. (h) Detached fragments of bands: all are of band 1 (valvocopula), each with a single row of round areolae; the upper two fragments are lying with the exterior uppermost and here the pars exterior appears rough, though without discrete papillae. Scale bars = 2 μm.

**Figure S8.** *Tryblionella* cf. *gaoana*, isolate Tokiane4 HK533, and *T. gaoana*, isolate SZCZCH97. (a, b) *Tryblionella* cf. *gaoana*, valves, LM (all other images SEM). (c) *Tryblionella* cf. *gaoana*, valve exterior, with a tangle of narrow girdle bands to the right. (d) *Tryblionella* cf. *gaoana*. valve interior. (e, f) *T*. *gaoana* valves, for comparison with *Tryblionella* cf. *gaoana*. (g) *Tryblionella* cf. *gaoana*. Detail of disassembled frustule (the same specimen as in (c)), showing the valve apex and multiple bands in the cingulum. The open end of band 1 and the closed ends of bands 2, 4, and 6 (arrowheads) are visible at this pole. (h) *Tryblionella* cf. *gaoana*, center inside, overlain by part of band 1 (valvocopula). The fibulae are wide solid structures separated by small rounded portulae; the central portula is wider. The smooth pars interior of the valvocopula (**pi**) bears short projections corresponding to the valve transapical ribs; the pars exterior (**pe**) bears densely spaced papillae and a single, irregular row of poroids (e.g., arrows). (i) *Tryblionella* cf. *gaoana*, partly collapsed frustule. One of the valves (top) displays the proximal mantle, with short striae of three to five areolae, plus a row of areolae in the raphe canal (e.g., white arrows). Note the absence of a marginal ridge at the junction between the valve face and distal mantle (arrowhead) and the scattered small papillae on the valve face, especially distally. Scale bars = 5 μm (a, b) or 2 μm (c–i).

**Figure S9.** *Tryblionella plana* var. *fennica* (a) whole valve, exterior. (b) Center outside. Note reticulate thickenings on the axial sternum and a single row of poroids in the raphe canal (arrow). (c) Part inside: smooth inner face of the axial sternum and circular poroids. (d) Valve interior viewed from the proximal side, with porose conopeum (arrow); just below is a single row of poroids that open into the proximal side of the raphe canal. (e) Center inside, showing wider central interspace and ± riblike fibulae, some of which are single, others fused in groups of two or three. (f) Valve pole, outside, with sail‐like marginal ridge, proximally directed terminal raphe fissure, and a single line of poroids on the distal side of the raphe canal (cf. b). Note that in the proximal section of each stria, the poroids open externally into an elongate groove. Scale bars = 10 μm (a) or 2 μm (b–f).

**Figure S10.** *Tryblionella acuminata* SEM, the type species of *Tryblionella*, from brackish sediment near Ferrybridge, at the mouth of the Fleet lagoon, Dorset, England, SEM. These images (from a Cambridge S4 Stereoscan, operated at 20 kV, with c. 45° tilt) were taken before 1977 during PhD research by DGM; some were used in a PhD dissertation (Mann 1978), but those shown here have been digitized from the original negatives. (a) Valve interior. (b) Center, showing the raphe slit opening onto the crest of a narrow ridge and central raphe endings; note the biseriate striae. (c) Center, exterior: note the strongly undulate valve face and externally thickened transapical ribs interrupted by a plain axial sternum. (d) Valve interior with small fibulae (e.g., arrows), each connecting with a single transapical rib (virga). (e) Valve pole, exterior, with terminal fissure tightly hooked toward the proximal side. (f) Valve pole: note that the proximal mantle bears short striae of a few (apparently two) areolae (e.g., arrow).

**Figure S11.** Strict consensus tree of the 23 most parsimonious trees found in the phylogenetic analysis of *Tryblionella* and *Tryblionella*‐like taxa with available EM data, based on morphological characters of valves listed in Table 1. Each of the trees has one of the 12 characters in the dataset mapped by color, with the character states and corresponding color listed to the right of each tree.

**Table S1.** List of studies taxa and strains of *Nitzschia* s.l. and *Tryblionella* s.l.

**Table S2.** Chemical and physical parameters for the Polish strains of *Tryblionella apiculata* and *T. hungarica*.

**Appendix S1.** Material and Methods.
