# Supplementary figures and images for: Taxonomic review of Tryblionella with special reference to the Apiculatae group—New characters of genus Tryblionella sensu stricto (Bacillariaceae)
Source: J Phycol. 2025 Mar 18;61(2):330–52. doi: 10.1111/jpy.70004 (PMC12044406; doi:10.1111/jpy.70004)

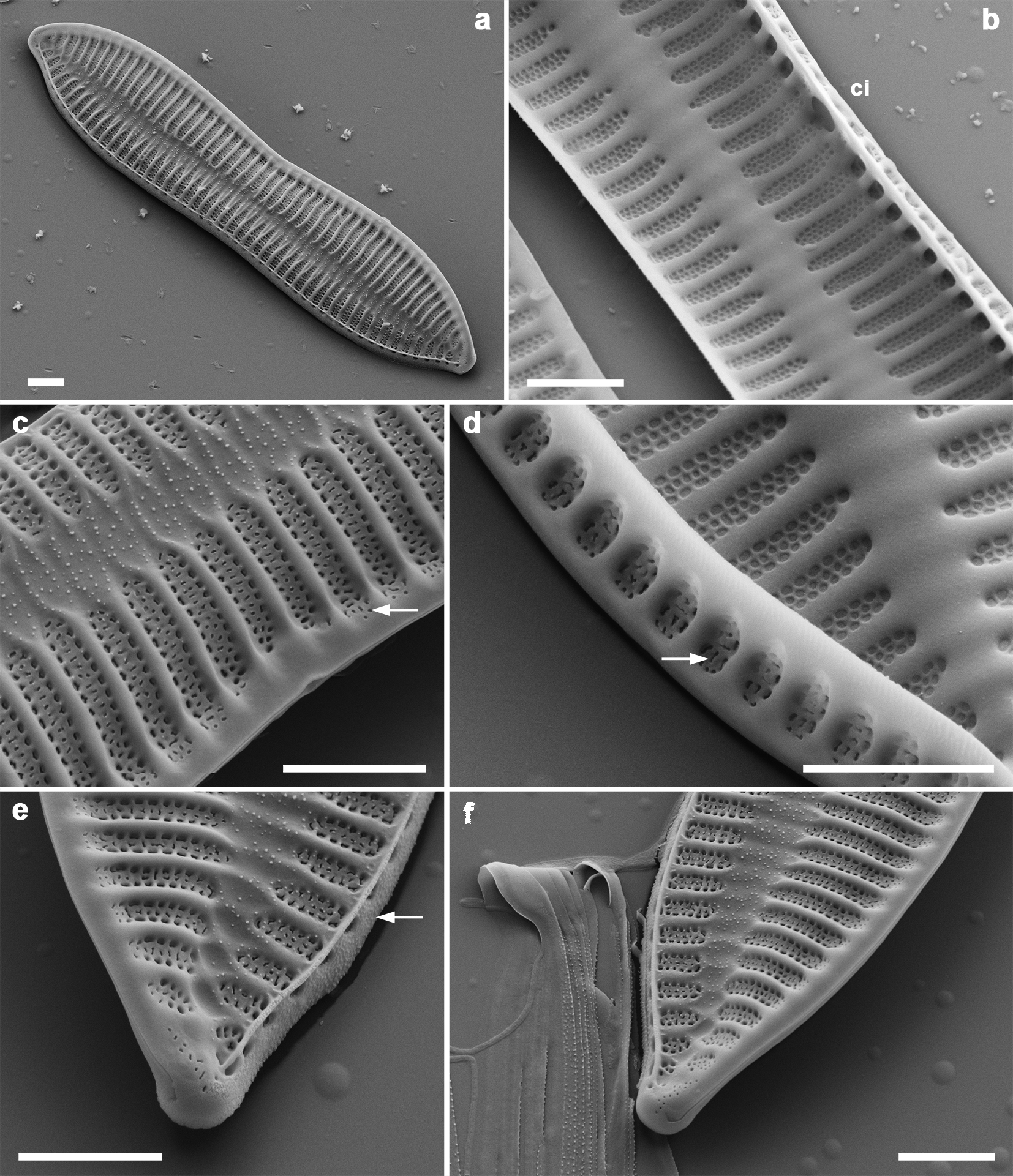

Supplement: Supplementary file 1 — Figure S1. Tryblionella apiculata, clone TRY946CAT, SEM. (a) Whole valve, exterior. (b) Valve interior, central part, showing the axial sternum interrupting the striae and the fibulae, which have a 1:1 relationship with the transapical ribs, except at the center; here, the relationship is lost, and there is a wider interspace (ci). (c) External detail of center. Note the notch in the raphe canal containing the central raphe endings, the multiseriate striae containing irregularly shaped poroids, the thickened transapical ribs (virgae), and small warts on the axial sternum. The striae continue into the wall of the raphe canal (arrow). (d) Valve mantle with very short multiseriate striae; at least part of each stria (e.g., at arrow) opens into the raphe canal. (e) The valve end with the terminal raphe fissure hooked toward the narrow (proximal) side of the valve. Note the small warts on the (distal) mantle (arrow), the small marginal ridge separating the distal mantle from the valve face, and the row of apparently simple poroids on the mantle just beneath the marginal ridge. (f) The valve end with the terminal raphe fissure hooked toward the wider (distal) side of the valve. Scale bars = 2 μm. [file JPY-61-330-s003.tif]

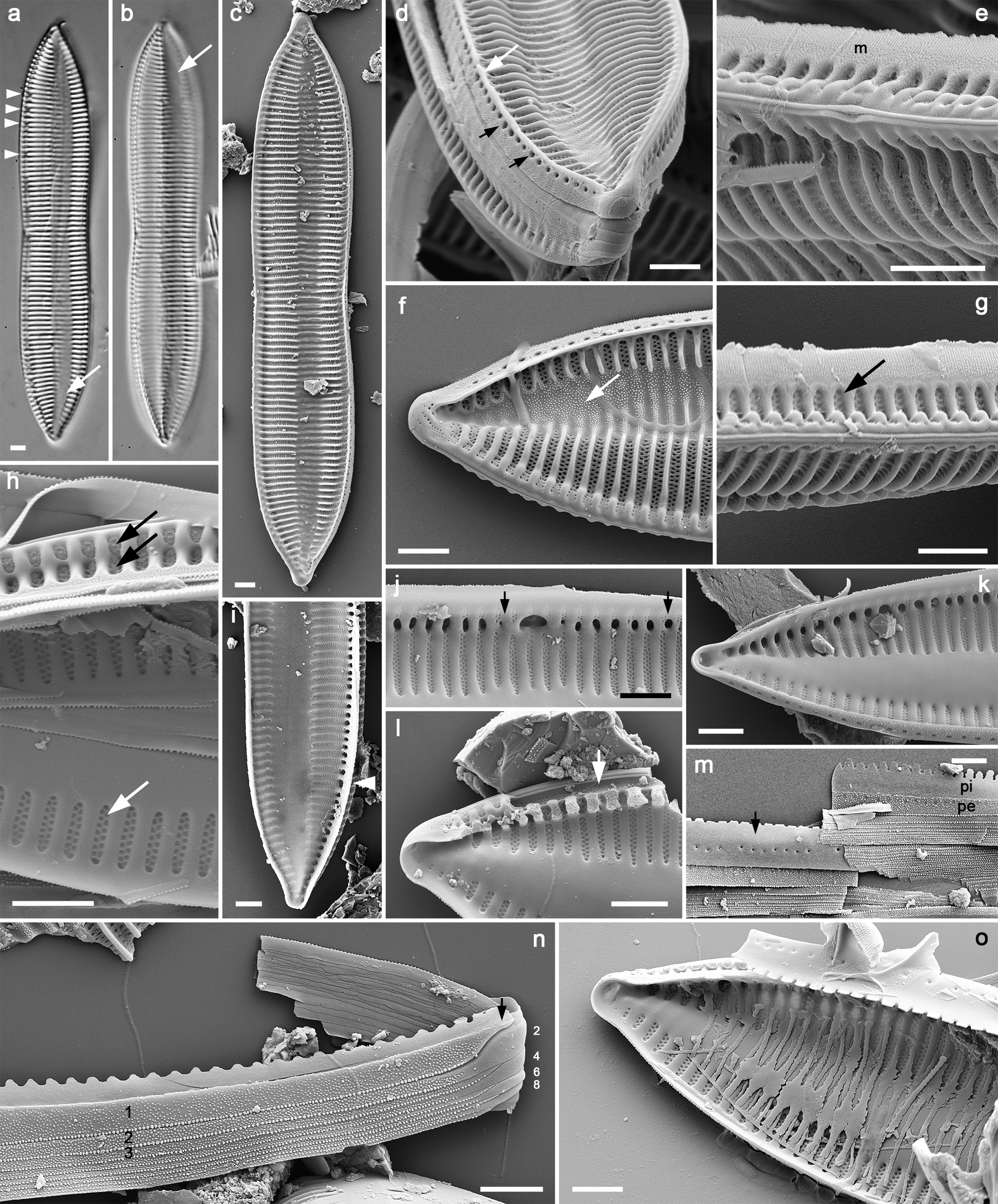

Supplement: Supplementary file 2 — Figure S2. Tryblionella apiculata, isolate FlMan33, (a–b) LM and (c–o) SEM. (a–b) Valves: raphe to the left: note the irregularities in the line of fibulae (e.g., arrowheads) and transapical striations that appear to traverse the whole valve face, except for short strips near the poles (arrows). (c) Calve exterior. (d) Frustule, highly tilted, showing a strongly undulate valve face and a marginal ridge (arrow) separating the valve face and distal mantle, and a single row of round areolae (e.g., arrows) along the mantle. (e) Center, with raised and keeled raphe and central raphe endings. (f) Valve pole, exterior, with biseriate striae interrupted by an axial sternum with a dense scatter of papillae. A plainer area is present near the poles (cf. arrows in (a–b)). (g) Oblique view of the proximal mantle with biseriate striae (e.g., arrow). (h) Proximal mantle with biseriate striae (black arrows) extending into the raphe canal wall (top arrow). Inside (below), the valve surface is smooth. Note that the distal striae (e.g., white arrow) are simple, with no sign of the extra mantle areolae present externally (cf. (d)). (i) Valve interior: fibulae generally spaced one per transapical rib, but sometimes getting out of phase (e.g., arrowhead). (j) Valve center inside with larger central portulae and an unusually large fibula (arrow). (k) Valve pole, inside. (l) Broken valve showing the raphe canal (arrow) and fibula structure. (m) Fragments of the girdle. Externally, all bands bear small papillae (see the pars exterior of band 1, pe) and band 1 bears a single row of small areolae. The pars interior (pi) and the internal face of band 1 (arrow) are smooth. (n) Detached cingulum with the open ends of bands 1 (arrow), 3, 5, 7, and 9, and the closed ends of bands 2, 4, 6 and 8 (numbered). The abvalvar margin of each band is marked externally by an orderly row of papillae. (o) Forming valve lying within the valve of the parent cell: transapical ribs beginning to fuse to form axial [file JPY-61-330-s001.tif]

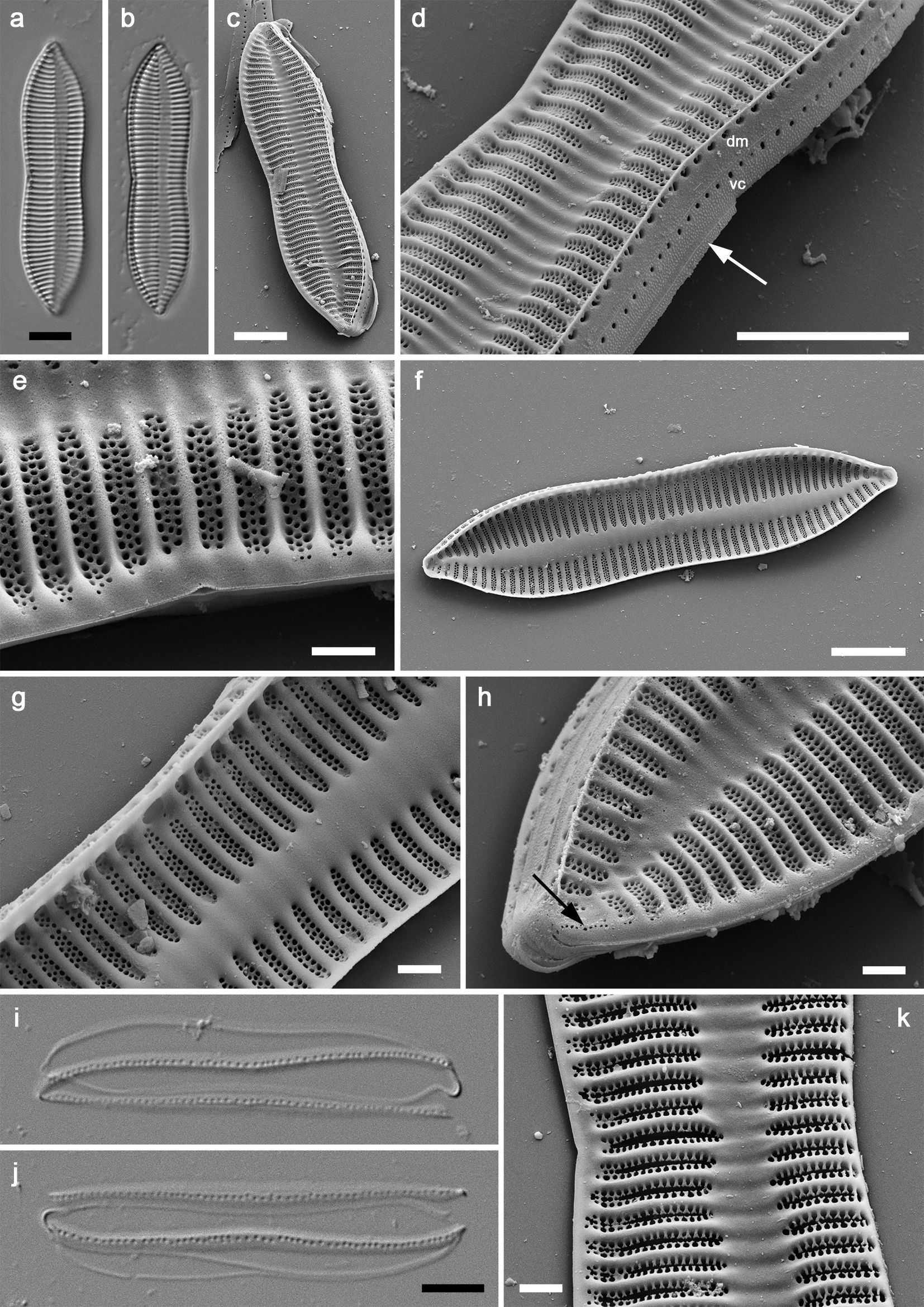

Supplement: Supplementary file 3 — Figure S3. Tryblionella apiculata, isolate s0863, LM (a, b, i, j) and SEM (c–h, k; all with 25° tilt). SEM indicates that the material was somewhat eroded, lacking occlusions in the areolae and with evidence of pitting in the silica basal layer. (a, b) Valves. (c) Valve exterior. (d) Theca, seen from the distal side. Note the presence of a marginal ridge separating the valve face from the distal mantle (dm), which contains short striae of triplet or doublet areolae (corresponding to the tri‐ or biseriate striae of the valve face). The first band (valvocopula, vc) is somewhat wider than the second (arrow). The mantle and girdle bands bear densely packed papillae. (e) Valve center outside. Note triseriate striae, which extend into the raphe canal and central raphe endings. (f) Valve interior: each transapical rib (virga) bears a fibula, except at the center (see g). (g) Valve center, inside. The center most portula is wider than the others. (h) Valve pole, outside, with distally curved terminal fissure and a distinctive row of poroids nearby (arrow) that do not relate to valve face striae. (i, j) Two examples of bands 1 and 2. Both bands are open. The first (valvocopula) bears a single row of areolae, while the second is much narrower and lacks areolae. (k) Stage in valve formation, in which the axial sternum is already complete, and the areolae on either side of the transapical ribs are almost completely delimited, but the third (central) row of areolae of the triseriate striae is only incipient. At this stage, the fibulae are still absent (the side of the valve corresponding to the proximal mantle has snapped off). Scale bars = 10 μm (a, b, i, j), 5 μm (c, d, f) or 1 μm (e, j, h, k). [file JPY-61-330-s012.tif]

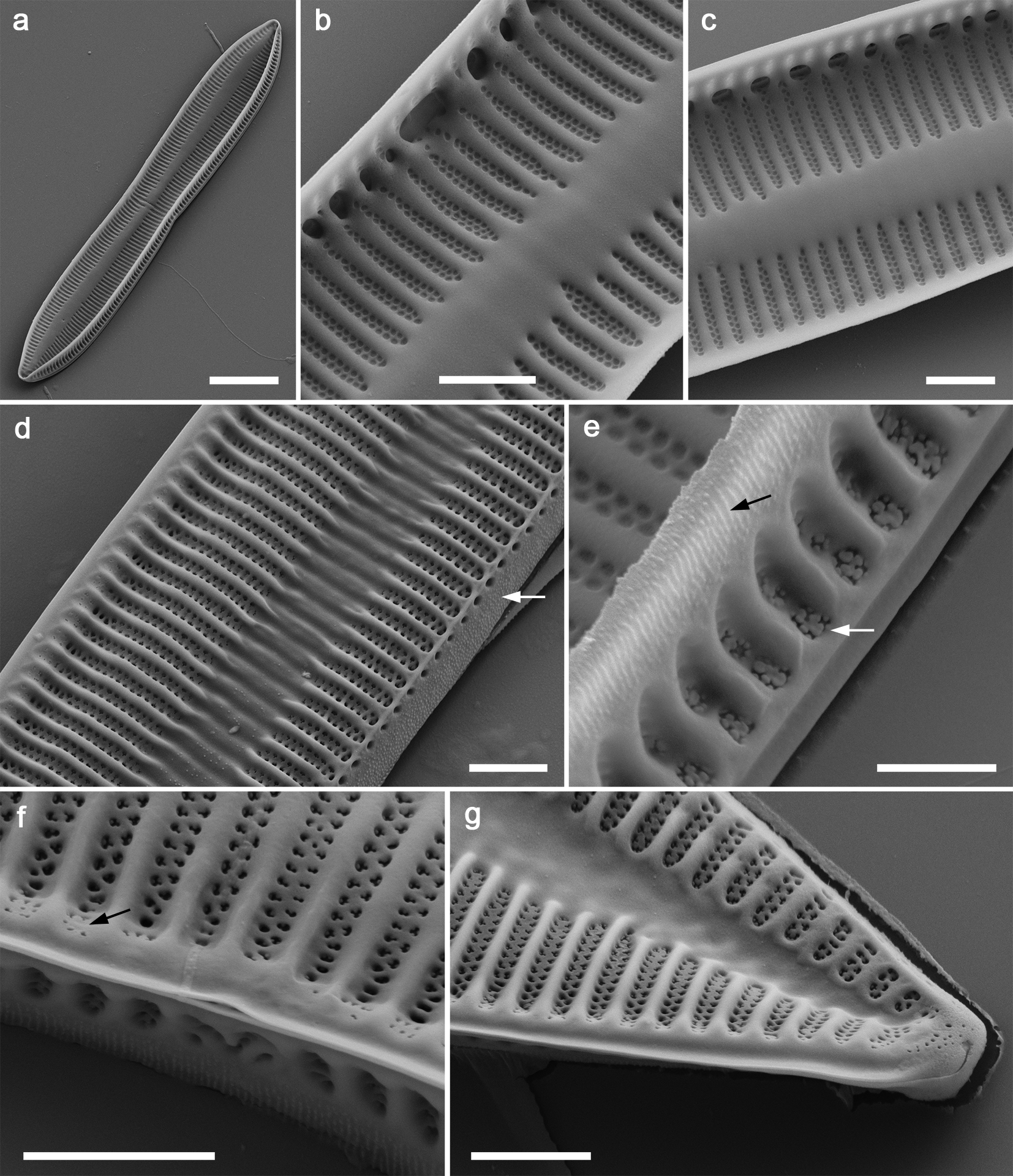

Supplement: Supplementary file 4 — Figure S4. Tryblionella hungarica, clone TRY981CAT, SEM. (a) Whole valve, interior. (b) Valve interior, central part, with wider central interspace (ci). (c) Valve interior, part showing the axial sternum interrupting the striae and the fibulae, which do not have an exact spatial relationship with the transapical ribs, there being many fewer fibulae. (d) Part of valve exterior, showing biseriate striae, axial sternum, thickened transapical ribs (virgae), small marginal ridge, a row of apparently simple poroids just beneath the marginal ridge, and small warts on the mantle. (e) Proximal mantle, showing lines of small warts extending to the valve margin (black arrow) and very short multiseriate striae, with at least part of each stria (e.g., white arrow) opening into the raphe canal. (f) External detail of center. The largely biseriate striae contain irregularly shaped poroids (often shaped like clover leaves) and continue into the wall of the raphe canal (e.g., at arrow), where the poroids become smaller and simpler. (g) The valve end with the terminal raphe fissure hooked toward the wider (distal) side of the valve. Note that the raphe opens onto the crest of a narrow ridge (see also (f)). Scale bars = 2 μm, except (a) (10 μm) and (e) (1 μm). [file JPY-61-330-s007.tif]

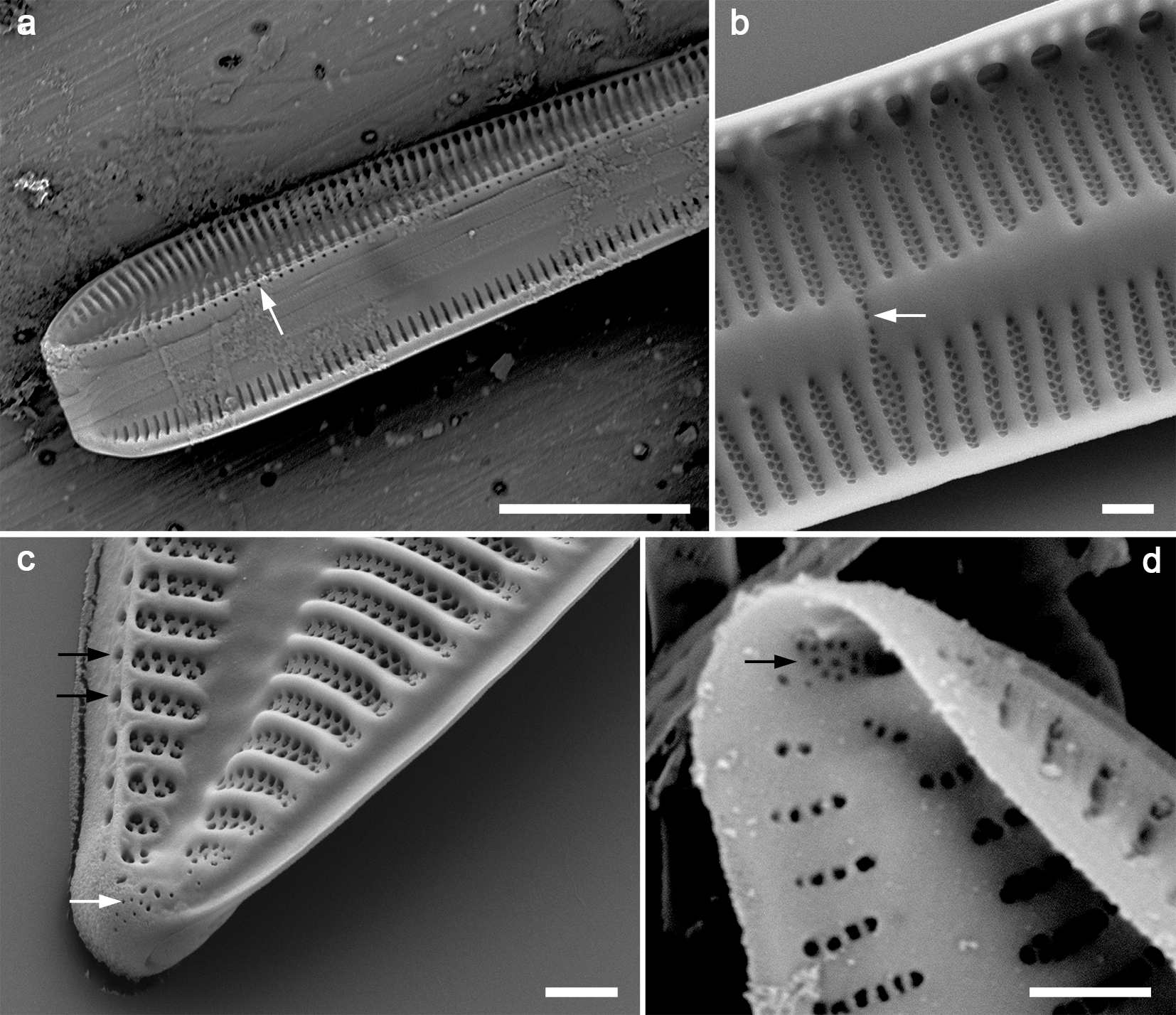

Supplement: Supplementary file 5 — Figure S5. Tryblionella hungarica SEM. (a, d) Strain SZCZ E683 and (b, c) strain TRY981CAT. (a) Intact frustule in girdle view, showing the girdle composed of several narrow bands and a row of single round areolae on the distal mantle (arrow), which lie just below a slim marginal ridge; note also the contrasting structure of the distal and proximal mantles. (b) Valve interior, center, showing a stria that bridges the sternum (arrow). (c) Valve pole, exterior. Note the single round areolae on the distal mantle (e.g., black arrows) and a group of poroids near the terminal fissure of the raphe that resembles a pore field (white arrow). (d) Interior of valve pole, showing the group of closely spaced small pores in the “pore field” (arrow). Scale bars = 10 μm (a) or 1 μm (b–d). [file JPY-61-330-s013.tif]

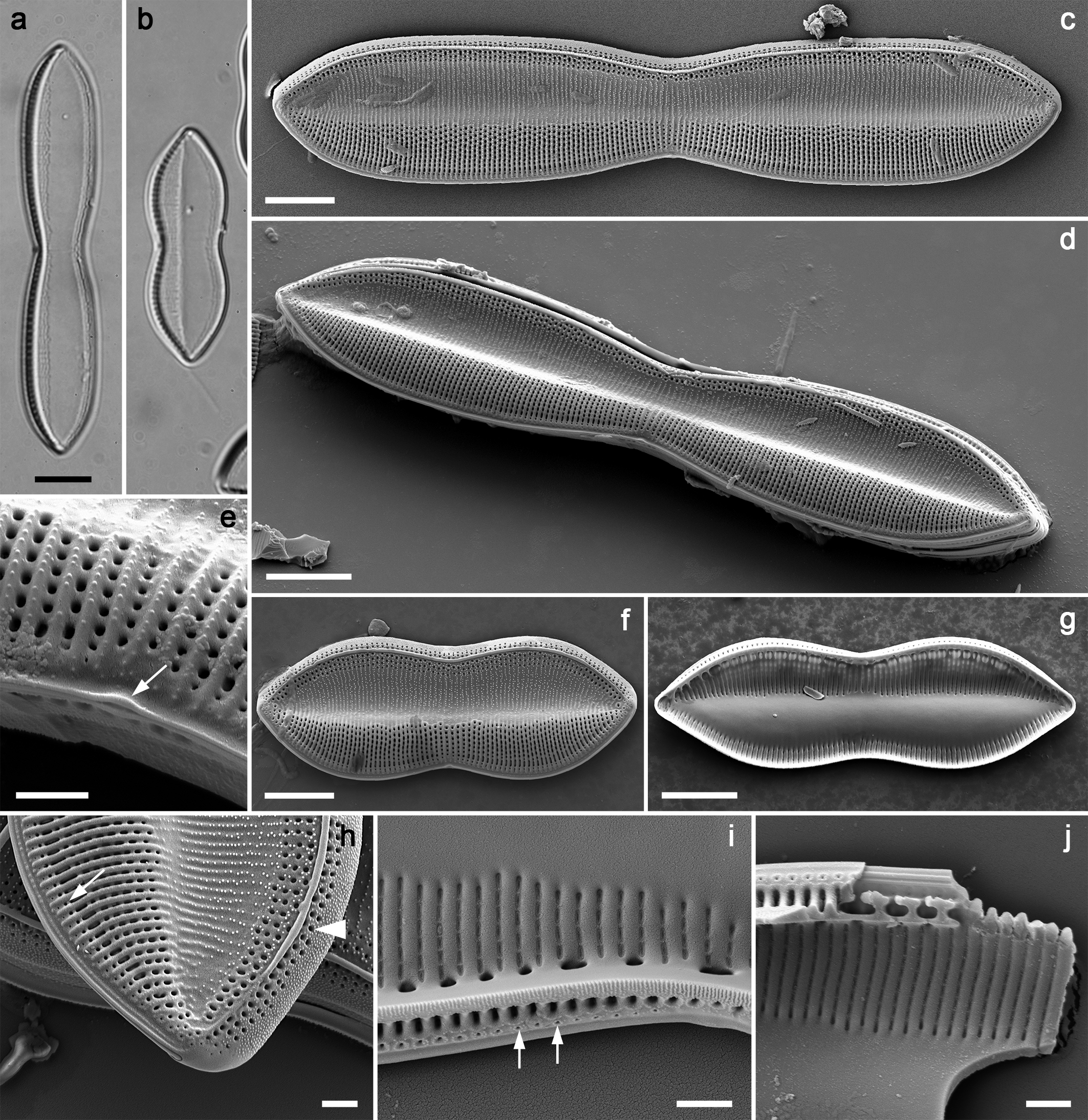

Supplement: Supplementary file 6 — Figure S6. Tryblionella marginulata (a–b) LM, (c–j) SEM. (b, f–h) Isolates PDK38 and (a, c–e, i–j) PDK142 LM. (a–b) Valves. (c) Whole valve, exterior, zero tilt. (d) Frustule tilted; note the prominent fold of the valve face. (e) Detail of the valve center in (c) with central raphe endings (arrow). (f–g) External and internal views of valves. (h) The valve pole tilted. Note the double row of areolae along the raphe canal (arrow), the scatter of small papillae on the valve face and mantle, and the presence of a marginal ridge, below which are two or three areolae (e.g., arrowhead) in each stria. (i) Oblique view of the interior of the proximal half of the valve, showing the fibulae and a single row of areolae (e.g., arrows) opening into the proximal wall of the raphe canal. (j) Fractured valve; note the complex structure of the fibulae. Scale bars = 10 μm (a–b), 5 μm (c–f), or 1 μm (e, h–j). [file JPY-61-330-s002.tif]

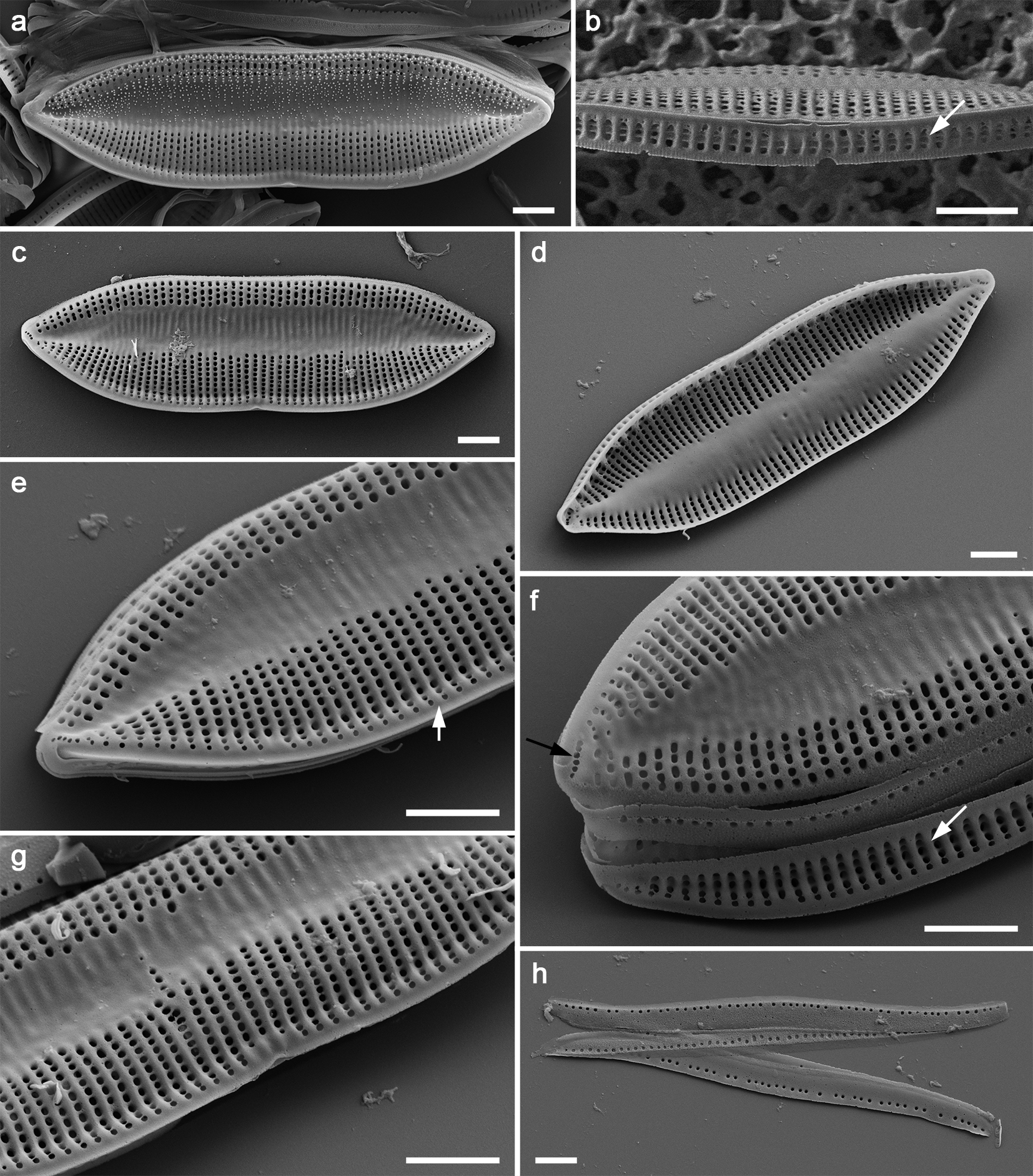

Supplement: Supplementary file 7 — Figure S7. Tryblionella spp., SEM (25° tilt, unless stated otherwise). (a) Tryblionella sp. isolate Tokiane4 HK533, whole valve exterior, zero tilt, for comparison with (b) T. gaoana, isolate SZCZCH97 (see Witkowski et al. 2016), highly tilted: proximal mantle with short striae of 3 areolae (e.g., arrow). (c–h) T. gaoana isolate TA426. (c) Whole valve, exterior, zero tilt. (d) Valve interior. (e) Valve pole, with terminal fissure curving toward the distal side and raphe opening onto the crest of a narrow ridge. Two poroids in each stria open into the raphe canal (arrow). (f) Frustule pole. Note the absence of a marginal ridge and the presence of a row of special (non‐stria) areolae adjacent to the terminal fissure (black arrow); short striae of 3–5 areolae are present on the proximal mantle (e.g., white arrow). (g) Valve center outside. (h) Detached fragments of bands: all are of band 1 (valvocopula), each with a single row of round areolae; the upper two fragments are lying with the exterior uppermost and here the pars exterior appears rough, though without discrete papillae. Scale bars = 2 μm. [file JPY-61-330-s006.tif]

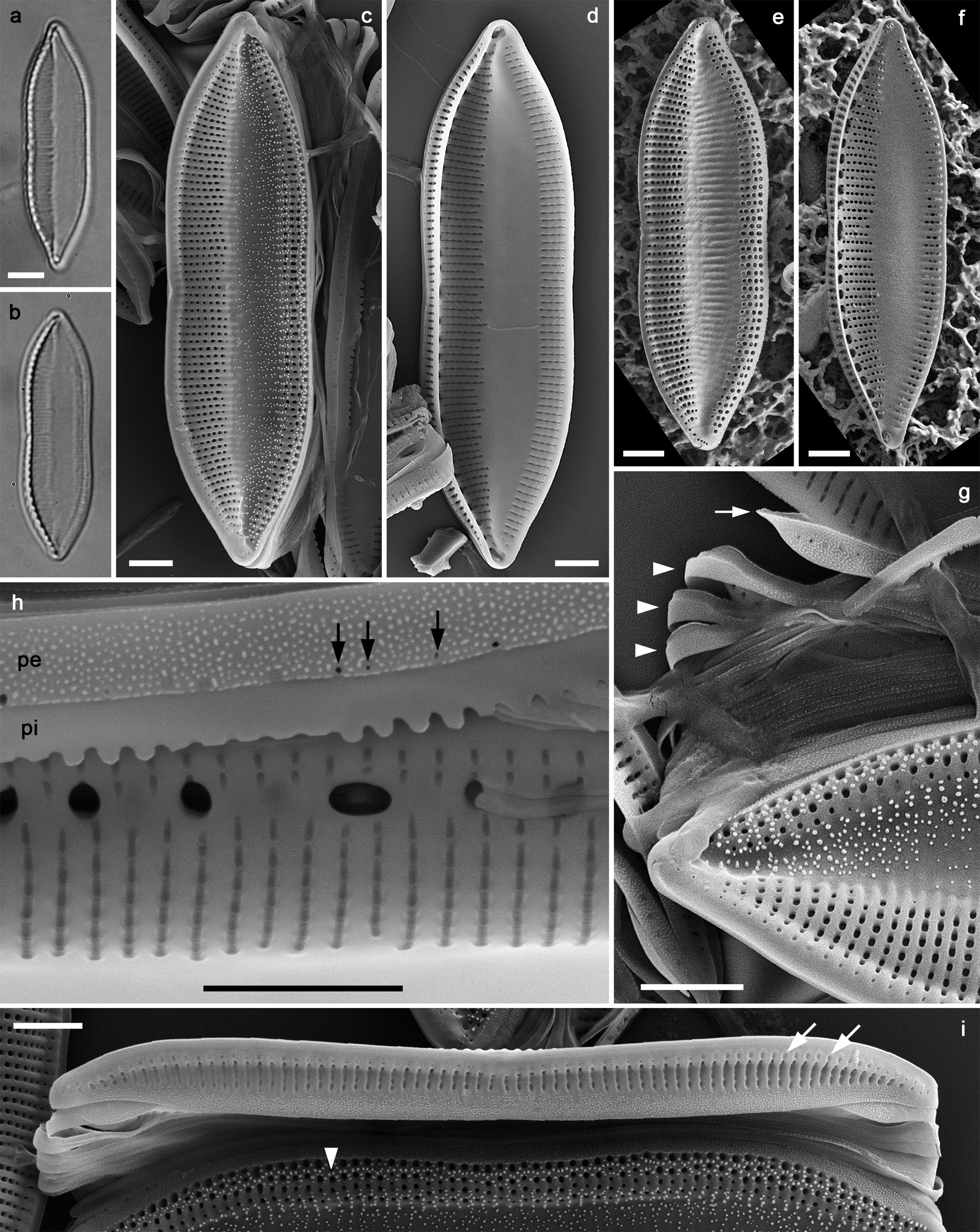

Supplement: Supplementary file 8 — Figure S8. Tryblionella cf. gaoana, isolate Tokiane4 HK533, and T. gaoana, isolate SZCZCH97. (a, b) Tryblionella cf. gaoana, valves, LM (all other images SEM). (c) Tryblionella cf. gaoana, valve exterior, with a tangle of narrow girdle bands to the right. (d) Tryblionella cf. gaoana. valve interior. (e, f) T. gaoana valves, for comparison with Tryblionella cf. gaoana. (g) Tryblionella cf. gaoana. Detail of disassembled frustule (the same specimen as in (c)), showing the valve apex and multiple bands in the cingulum. The open end of band 1 and the closed ends of bands 2, 4, and 6 (arrowheads) are visible at this pole. (h) Tryblionella cf. gaoana, center inside, overlain by part of band 1 (valvocopula). The fibulae are wide solid structures separated by small rounded portulae; the central portula is wider. The smooth pars interior of the valvocopula (pi) bears short projections corresponding to the valve transapical ribs; the pars exterior (pe) bears densely spaced papillae and a single, irregular row of poroids (e.g., arrows). (i) Tryblionella cf. gaoana, partly collapsed frustule. One of the valves (top) displays the proximal mantle, with short striae of three to five areolae, plus a row of areolae in the raphe canal (e.g., white arrows). Note the absence of a marginal ridge at the junction between the valve face and distal mantle (arrowhead) and the scattered small papillae on the valve face, especially distally. Scale bars = 5 μm (a, b) or 2 μm (c–i). [file JPY-61-330-s008.tif]

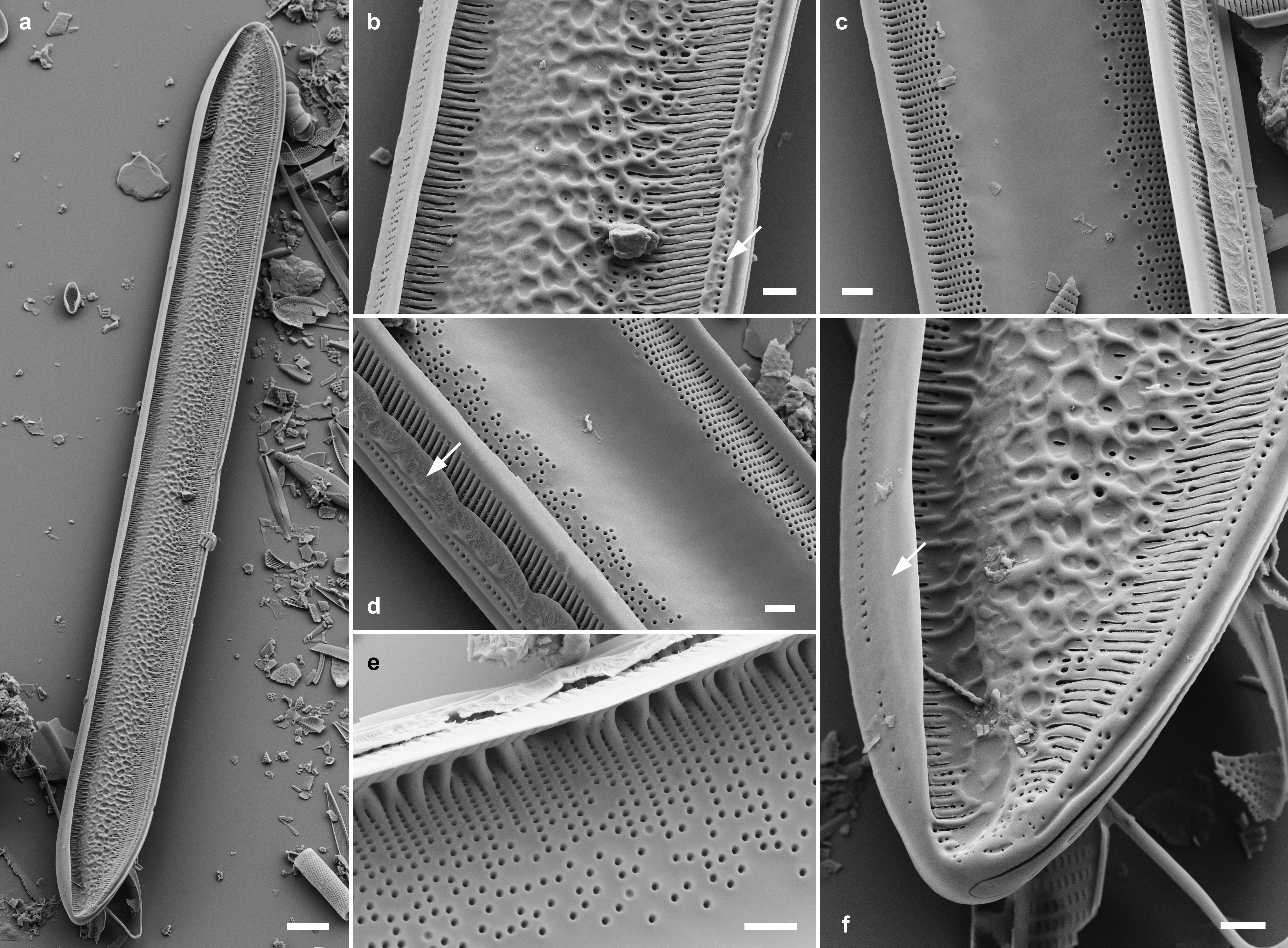

Supplement: Supplementary file 9 — Figure S9. Tryblionella plana var. fennica (a) whole valve, exterior. (b) Center outside. Note reticulate thickenings on the axial sternum and a single row of poroids in the raphe canal (arrow). (c) Part inside: smooth inner face of the axial sternum and circular poroids. (d) Valve interior viewed from the proximal side, with porose conopeum (arrow); just below is a single row of poroids that open into the proximal side of the raphe canal. (e) Center inside, showing wider central interspace and ± riblike fibulae, some of which are single, others fused in groups of two or three. (f) Valve pole, outside, with sail‐like marginal ridge, proximally directed terminal raphe fissure, and a single line of poroids on the distal side of the raphe canal (cf. b). Note that in the proximal section of each stria, the poroids open externally into an elongate groove. Scale bars = 10 μm (a) or 2 μm (b–f). [file JPY-61-330-s004.tif]

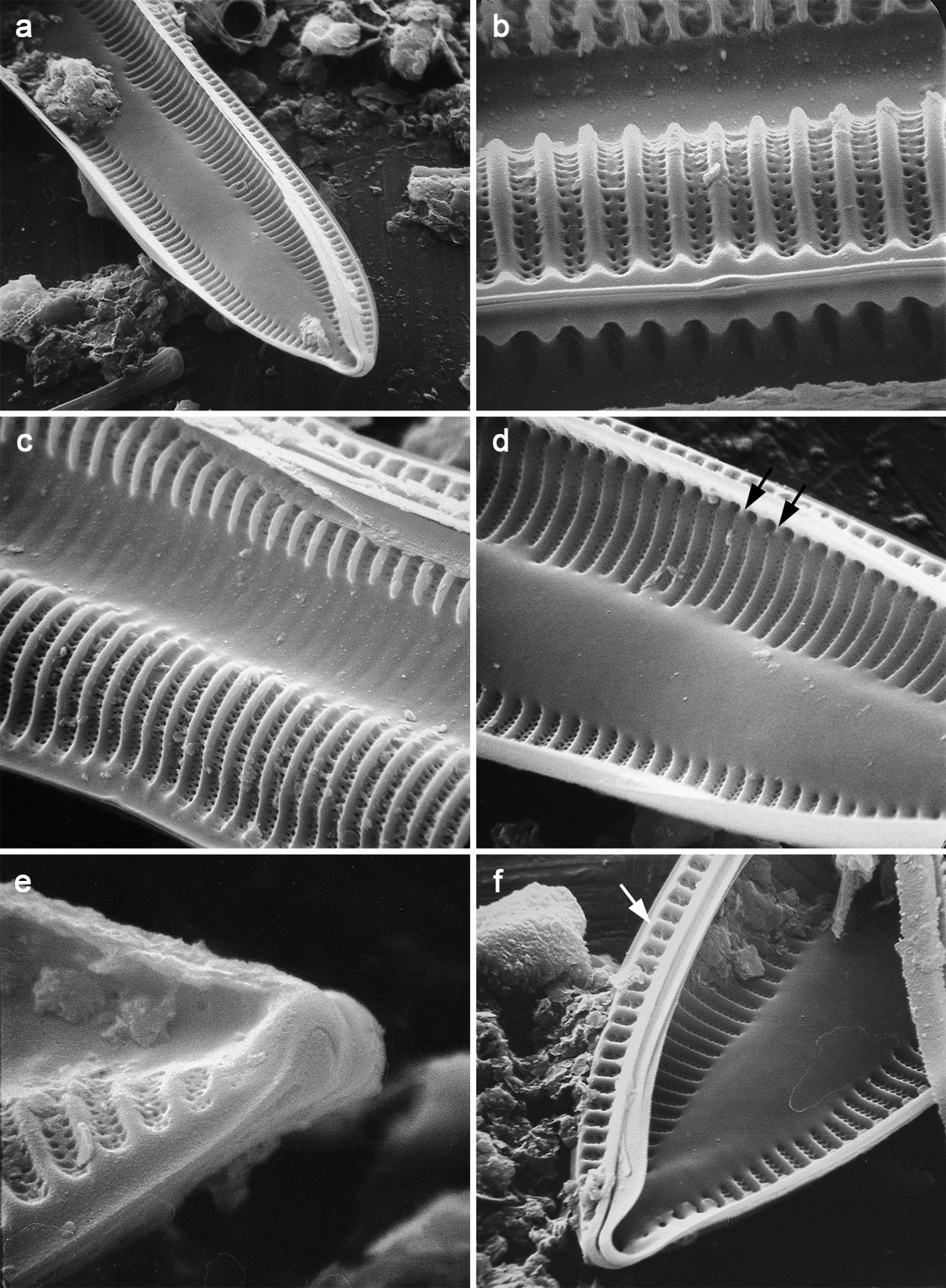

Supplement: Supplementary file 10 — Figure S10. Tryblionella acuminata SEM, the type species of Tryblionella, from brackish sediment near Ferrybridge, at the mouth of the Fleet lagoon, Dorset, England, SEM. These images (from a Cambridge S4 Stereoscan, operated at 20 kV, with c. 45° tilt) were taken before 1977 during PhD research by DGM; some were used in a PhD dissertation (Mann 1978), but those shown here have been digitized from the original negatives. (a) Valve interior. (b) Center, showing the raphe slit opening onto the crest of a narrow ridge and central raphe endings; note the biseriate striae. (c) Center, exterior: note the strongly undulate valve face and externally thickened transapical ribs interrupted by a plain axial sternum. (d) Valve interior with small fibulae (e.g., arrows), each connecting with a single transapical rib (virga). (e) Valve pole, exterior, with terminal fissure tightly hooked toward the proximal side. (f) Valve pole: note that the proximal mantle bears short striae of a few (apparently two) areolae (e.g., arrow). [file JPY-61-330-s009.tif]

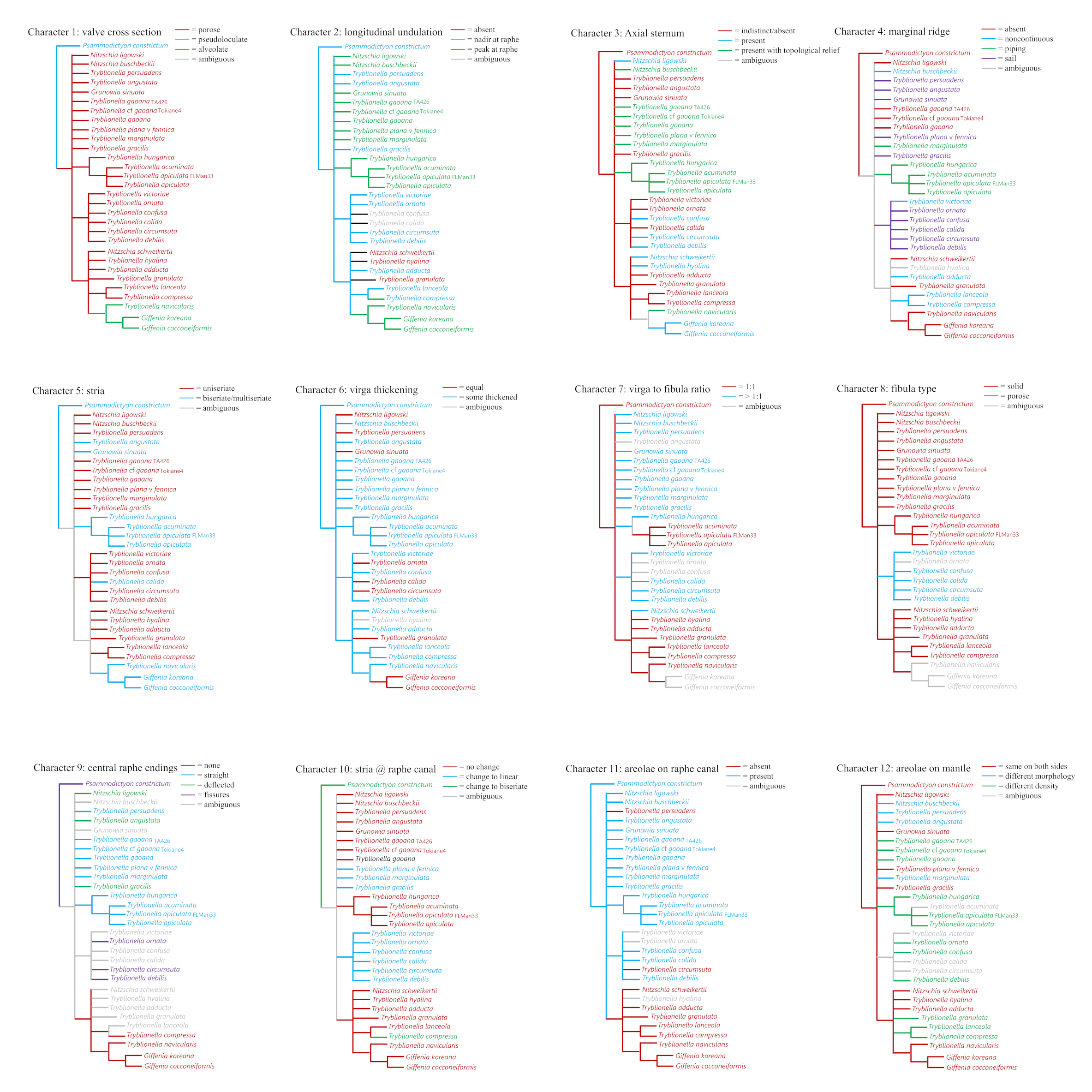

Supplement: Supplementary file 11 — Figure S11. Strict consensus tree of the 23 most parsimonious trees found in the phylogenetic analysis of Tryblionella and Tryblionella‐like taxa with available EM data, based on morphological characters of valves listed in Table 1. Each of the trees has one of the 12 characters in the dataset mapped by color, with the character states and corresponding color listed to the right of each tree. [file JPY-61-330-s014.jpg]
