# Supplementary material for: Taxonomic review of Tryblionella with special reference to the Apiculatae group—New characters of genus Tryblionella sensu stricto (Bacillariaceae)
Source: J Phycol. 2025 Mar 18;61(2):330–52. doi: 10.1111/jpy.70004 (PMC12044406; doi:10.1111/jpy.70004)
Supplement: Supplementary file 12 — Table S1. List of studies taxa and strains of Nitzschia s.l. and Tryblionella s.l. [file JPY-61-330-s010.docx]

| Taxa/Original identification | Strain | Sampling location | | | Data source |
| --- | --- | --- | --- | --- | --- |
| *Tryblionella hungarica* | SZCZ E2191 | Baltic Sea, West Pomeranian Voivodeship, Poland | 54°5'59.17'' N 15°5'28.72'' E | Planktonic, the Baltic Sea littoral zone | This study |
| *T. hungarica* | SZCZ E683 | Pełczyska, Łódź Voivodeship, Poland | 51°58'35.68'' N 19°14'17.02'' E | Benthic, the silty shore of the pond | This study |
| *T. hungarica* | Try986CAT | IRTA aquaculture lagoon, Ebro Delta, Catalonia, Spain | 40°37'38.88'' N 0°39'39.26'' E | Benthic, lagoon sediment | Mann et al. (2021) |
| *T. hungarica* | Try981CAT | IRTA aquaculture lagoon, Ebro Delta, Catalonia, Spain | 40°37'38.88'' N 0°39'39.26'' E | Benthic, lagoon sediment | Mann et al. (2021) |
| *T. apiculata* | Try946CAT | IRTA aquaculture lagoon, Ebro Delta, Catalonia, Spain | 40°37'38.88'' N 0°39'39.26'' E | Benthic, lagoon sediment | Mann et al. (2021) |
| *T. apiculata* | s0863 | Buda Island, Ebro Delta, Catalonia, Spain | 40°41'60.00'' N 0°52'0.00'' E | Benthic, lagoon sediment | Mann et al. (2021) |
| *T.* *apiculata* | FLMan33 panduriformA23 | Crystal River, Florida, USA | 28°53'27'' N 82°35'49'' W | Epizooic, manatee skin, oligohaline waters | Frankovich et al. (2018) |
| *Nitzschia gaoana* | TA426 | Geunso Bay, Taean, South Korea | 36°44'12.06" N 126°10'47.52" E | Benthic, tidal flats | An et al. (2017) |
| *Nitzschia* sp. | Tokiane4 nitzG1 | Toliara, the Atsimo-Andrefana region, Madagascar | N/D | N/D | Lobban et al. (2021) |
| *Tryblionella marginulata* | RebReef3_  elongpeanutPDK142 | Rebotel Reef, Palau | 7°14′55.8" N, 134°14′8.9" E | Benthic, coral sand (5-10 m depth) | This study |
| *T. marginulata* | RebReef2_  pandurifPDK38 | Rebotel Reef, Palau | 7°14′55.8" N, 134°14′8.9" E | Benthic, coral sand (5-10 m depth) | This study |
| *T. debilis* | BC0502 | Artificial stream, Royal Botanic Garden Edinburgh, Edinburgh, Scotland, UK | 55°57′51.24″ N  3°12′18.71″ W | Periphyton | Mann et al. (2021) |
| *Giffenia cocconeiformis* | – | Ngwenyana River, Eastern  Cape Province, South Africa | N/D | Periphytic from piers of the bridge in strongly brackish water | Giffen (1963) and Round and Basson (1997) |
| *G. koreana* | – | Suncheonman Bay Wetland, South Korea | 34°52′10.12″ N 127°29′27.56″ E | Core samples collected at a depth of 2.5 m in the tidal flat | Lee et al. (2023) |
| *Grunowia sinuata* | – | Afon Brân, Llandovery, Dyfed, UK | N/D | Epilithic, scratched from the underside of the bridge arch | Mann (1984) |
| *Nitzschia buschbeckii* | – | Davis Sea, Station Mirny, Island of Stroitelei | N/D | Epilithic, from the  rock sublittoral | Witkowski et al. (2004) |
| *N. lanceola* | – | Ngwenyana River, Eastern  Cape Province, South Africa | N/D | Periphytic from piers of the bridge in strongly brackish water | Giffen (1963) and Round and Basson (1997) |
| *N. ligowski* | – | Victoria Docks in Hull, UK | N/D | N/D | Witkowski et al. (2004) |
| *N. schweikertii* | – | Tiwi Beach, Kenya, Indian Ocean | 4°14' S  39°36' E | Epipsammic, coral sand | Witkowski et al. (2004) |
| *Psammodictyon constrictum* | – | Portobello, Edinburgh, UK | N/D | N/D | Mann in prep |
| *Tryblionella acuminata* | – | Isle of Portland, Dorset, Ferry bridge, UK | N/D | Various sediment from saltmarsh | Mann (1978) |
| *T. adducta* | – | The coasts of the Yellow Sea, China and South Korea | Data in Im et al. (2020) | Benthic, surface sediments | Im et al. (2020) |
| *T. angustata* | – | River Dee, Braemar, UK | N/D | Sediment | Mann (1984) |
| *T. calida* | – | Patos Lagoon estuary, State of Rio Grande do Sul, Brazil | 31º50'-32º09' S  52º00'-52º15' W | Periphytic and epiphytic | Bertolli et al. (2020) |
| *T. circumsuta* | – | Patos Lagoon estuary, State of Rio Grande do Sul, Brazil | 31º50'-32º09' S  52º00'-52º15' W | Periphytic and epiphytic | Bertolli et al. (2020) |
| *T. compressa* | Try1006CAT and Try1007CAT | Alfacs, Ebro Delta, Catalonia, Spain | 40°35'33.0" N 0°42'35.9" E | Coastal marsh sediment | Mann and Trobajo (2025) |
| *T. confusa* | – | Patos Lagoon estuary, State of Rio Grande do Sul, Brazil | 31º50'-32º09' S  52º00'-52º15' W | Periphytic and epiphytic | Bertolli et al. (2020) |
| *T. gracilis* | – | Ditch, Berkeley, Gloucestershire, UK | 51°41'10" N, 002°27'36" W | Sediment from drainage dich beside the Little Avon River | Mann (1984) |
| *T. granulata* | – | Patos Lagoon estuary, State of Rio Grande do Sul, Brazil | 31º50'-32º09' S  52º00'-52º15' W | Periphytic and epiphytic | Bertolli et al. (2020) |
| *T. hyalina* | – | The coasts of the Yellow Sea, China and South Korea | Data in Im et al. (2020) | Surface sediments | Im et al. (2020) |
| *T. navicularis* | – | River Avon, Cumberland Basin, Bristol, UK | N/D | Sediment | Mann (1978), Guttinger (1989) |
| *T. ornata* | – | Patos Lagoon estuary, State of Rio Grande do Sul, Brazil | 31º50'-32º09' S  52º00'-52º15' W | Periphytic and epiphytic | Bertolli et al. (2020) |
| *T. persuadens* | – | Cachoeira River, Eastern  Basin, Bahia state, Brazil | 14°47'14.24" S 39°16'10.12" W | Planktonic and periphytic | Cavalcante et al. (2013) |
| *T. plana* var. *fennica* | – | Lake of Menteith, Stirlingshire, Scotland, UK | 56°10'51" N  004°17'3.5" W | Epipelon, littoral zone circa 1m depth | This study |
| *T. victoriae* | – | Patos Lagoon estuary, State of Rio Grande do Sul, Brazil | 31º50'-32º09' S  52º00'-52º15' W | Periphytic and epiphytic | Bertolli et al. (2020) |

N/D – no data

An, S. M., Choi, D. H., Lee, J. H., Lee, H., & Noh, J. H. (2017). Identification of benthic diatoms isolated from the eastern tidal flats of the Yellow Sea: Comparison between morphological and molecular approaches. *PLoS One*, *12*, Article e0193972e0179422. https://doi.org/10.1371/journal.pone.0179422

Bertolli, L. M., Talgatti, D. M., Nascimento, T. M. da S., & Torgan, L. C. (2020). The genus *Tryblionella* W. Smith (Bacillariaceae, Bacillariophyta) in southern Brazil salt marshes. *Biota Neotrop*, *20*, Article e20190774. https://doi.org/10.1590/1676-0611-BN-2019-0774

Cavalcante, K. P., Tremarin, P. I., Freire, E. G., & Ludwig, T. A. V. (2013). *Tryblionella persuadens comb. nov.* (Bacillariaceae, Diatomeae): new observations on frustule morphology of a seldom recorded diatom. *An Acad Bras Cienc*, *85*, Article 1419–26. https://doi.org/10.1590/0001-37652013108112

Frankovich, T. A., Ashworth, M. P., Sullivan, M. J., Theriot, E. C., & Stacy, N. I. (2018). Epizoic and Apochlorotic *Tursiocola* species (Bacillariophyta) from the Skin of Florida Manatees (*Trichechus manatus latirostris*). *Protist*, *169*, 539–68. https://doi.org/10.1016/j.protis.2018.04.002

Giffen, M.H. 1963. Contributions to the diatom flora of South Africa - i. Diatoms of the estuaries of the Eastern Cape province. *Hydrobiologia*. 21:201–65.

Guttinger, W. (1989). Collection of SEM-micrographs of diatoms. Series 1–3.

Im, A., Khim, J. S., & Park, J. (2020). Taxonomy and distribution of two small *Tryblionella* (Bacillariophyceae) species from the Northeast Asian tidal flats. *Journal of Species Research*, *9*, 191–7. https://doi.org/10.12651/JSR.2020.9.3.191

Lee, S. D., Park, M., Lee, J. Y., Lee, H., & Choi, J. M. (2023). *Giffenia koreana* sp. nov. (Bacillariophyta): A newly identified epipelic diatom in the tidal flat sediments of Suncheonman Bay, extant for over 1, 400 Years. *Phytotaxa*, *629*, 187–96. https://doi.org/10.11646/phytotaxa.629.3.1

Lobban, C. S., Majewska, R., Ashworth, M., Bizsel, N., Bosak, S., Kooistra, W. H. C. F., Lam, D. W., Navarro, J. N., Pennesi, C., Sato, S., Van de Vijver, B., & Witkowski, A. (2021). Diatom Genus *Hyalosira* (Rhabdonematales emend.) and Resolution of its Polyphyly in Grammatophoraceae and Rhabdonemataceae with a New Genus, Placosira, and Five New *Hyalosira* Species. *Protist* *172*(3), Article 125816. https://doi.org/10.1016/j.protis.2021.125816

Mann, D. G. (1978). Studies in the Nitzschiaceae (Bacillariophyta). Ph.D. Dissertation, 1–386. University of Bristol.

Mann, D. G. (1984). An ontogenetic approach to diatom systematics. In *Proceedings of the 7th International Diatom Symposium*, edited by D. G. Mann. 113–44. O. Koeltz, Koenigstein.

Mann, D. G., Trobajo, R., Sato, S., Li, C., Witkowski, A., Rimet, F., Ashworth, M. P., Hollands, R. M., & Theriot, E. C. (2021). Ripe for reassessment: A synthesis of available molecular data for the speciose diatom family Bacillariaceae. *Molecular Phylogenetics and Evolution*, *158*, Article 106985. https://doi.org/10.1016/j.ympev.2020.106985

Round, F. E., & Basson, P. W. (1997). A new diatom genus (*Giffenia*) based on *Nitzschia cocconeiformis* Grun. and a note on *Nitzschia* (*Tryblionella*) *lanceola* Grun. *Diatom Research*, *12*(2), 347–55. https://doi.org/10.1080/0269249X.1997.9705426

Witkowski, A., Lange-Bertalot, H., Patrick Kociolek, J., Ruppel, M., Wawrzyniak-Wydrowska, B., Bak, M., & Brzezińska, A. (2004). Four new species of *Nitzschia* sect. Tryblionella (Bacillariophyceae) resembling *N. parvula*. *Phycologia*, *43*(5), 579–95. https://doi.org/10.2216/i0031-8884-43-5-579.1
