# Supplementary material for: Taxonomic review of Tryblionella with special reference to the Apiculatae group—New characters of genus Tryblionella sensu stricto (Bacillariaceae)
Source: J Phycol. 2025 Mar 18;61(2):330–52. doi: 10.1111/jpy.70004 (PMC12044406; doi:10.1111/jpy.70004)
Supplement: Supplementary file 13 — Table S2. Chemical and physical parameters for the Polish strains of Tryblionella apiculata and T. hungarica. [file JPY-61-330-s011.docx]

|  | Pełczyska pond | | | | Baltic sea |
| --- | --- | --- | --- | --- | --- |
|  | 10.2014 | 12.2014 | 03.2015 | 06.2015 | 06.2022 |
| pH | 9.1 | 8.8 | 8.5 | 9.6 | 8.0 |
| Conductivity [µS · cm^-1^] | 2788 | 3858 | 2645 | 5150 | N/A |
| Salinity [‰] | N/A | N/A | N/A | N/A | 7.08 |
| T [ᵒc] | 14.6 | 1.1 | 13.2 | 17.3 | 15.1 |
| HCO_3_^-^ [mg · L^-1^] | 296 | 451 | 320 | 284 | N/A |
| CO_2_^[HCO3-]^ [mg · L^-1^] | 107 | 163 | 112 | 102 | N/A |
| Cl^-^ [mg · L^-1^] | 716 | 912 | 1090 | 1524 | N/A |
| N_NH4_ [mg · L^-1^] | 0 | 0 | 0.05 | 0.02 | N/A |
| NH_4_^+^ [mg · L^-1^] | 0 | 0 | 0.06 | 0.03 | N/A |
| PO_4_^3-^ [mg · L^-1^] | 4.04 | 4.52 | 0.85 | 1.47 | N/A |
| P_PO4_ [mg · L^-1^] | 1.33 | 1.49 | 0.28 | 0.49 | N/A |
| SO_4_^2-^ [mg · L^-1^] | 103 | 151 | 190 | 197 | N/A |
| S_SO4_ [mg · L^-1^] | 34 | 50 | 63 | 66 | N/A |
| COLOR [mgPt · dm^-3^] | 80 | 120 | 50 | 80 | N/A |
| Mn^3+^ [mg · L^-1^] | 0.20 | 0.25 | 0.13 | 0.03 | N/A |
| Fe^2+/3+^ [mg · L^-1^] | 0.26 | 0.05 | 0.05 | 0.24 | N/A |
| Mg^2+^ [mg · L^-1^] | 17 | 34 | 32 | 37 | N/A |
| Ca^2+^ [mg · L^-1^] | 82 | 138 | 131 | 140 | N/A |
| Na^+^ [mg · L^-1^] | 375 | 402 | 463 | 689 | N/A |
| K^+^ [mg · L^-1^] | 73 | 69 | 68 | 59 | N/A |

N/A – not analysed
