# Supplementary material for: Taxonomic review of Tryblionella with special reference to the Apiculatae group—New characters of genus Tryblionella sensu stricto (Bacillariaceae)
Source: J Phycol. 2025 Mar 18;61(2):330–52. doi: 10.1111/jpy.70004 (PMC12044406; doi:10.1111/jpy.70004)
Supplement: Supplementary file 14 — Appendix S1. Material and Methods. [file JPY-61-330-s005.docx]

**Material &Methods**

*SZCZ samples (E683 & E2191)*

Sampling SZCZ

Benthic samples from Pełczyska and planktonic samples from the Baltic Sea were brought to the laboratory and the living material was enriched with f/2 culture medium (Guillard, 1975). Single cells of *T. hungarica* were isolated with a glass micropipette using an inverted light microscope Nikon Eclipse TS100 (Nikon, Tokyo, Japan) and cultivated to obtain monocultures, which were later included in the Szczecin Diatom Culture Collection (SZCZ) at the University of Szczecin.

DNA isolation & sequencing

To conduct phylogenetic analysis, the DNA was isolated from the established non-axenic monocultures of *Tryblionella hungarica* (SZCZ E683 from Pełczyska and SZCZ E2191 from the Baltic Sea coast). Three molecular markers SSU, *psbC*, *rbcL* (in case of E683) or only *rbcL* (in case of E2191) were amplified during PCR. Genomic DNA isolation was performed with a Chelex^®^ resin (cat. no. 142-2842-MSDS, Bio-Rad, Hercules, CA, USA): diatom strains were pelleted and 150 µL of 10% Chelex^®^ working solution was added to the strains in 1.5 mL Eppendorf tubes. After 20 minutes of heating at 95°C, the material was centrifuged for 5 minutes at 10,000 rpm (RCF = 6,010g). DNA-containing supernatant was put into fresh, sterile Eppendorf tubes and used for PCR.

*Phylogenetic analysis (molecular)*

A maximum likelihood analysis (ML) was performed using the concatenated three-gene dataset for 151 diatom taxa. The strain of *Triparma pacifica* (Guillou & Chrétiennot-Dinet), Ichinomiya & Lopes dos Santos, was chosen as an outgroup. Phylogenetic analyses were conducted with 1000 bootstrap replicates using ultra-fast bootstrap replications in IQ-TREE 2.2.0 (Nguyen et al., 2015; Minh et al., 2020). Prior to phylogenetic analyses sequences SZCZ E683 and E2191 were aligned with a reference database build using sequences available in GenBank with MAFFT 7 (Katoh & Standley, 2016) and trimmed with trimAl -automated1 option (Capella-Gutiérrez et al., 2009). The best models of evolution were evaluated on each alignment separately by ModelTest-NG (Darriba et al., 2020; Flouri et al., 2015) and datasets were concatenated with Phyutility software (Smith & Dunn, 2008). The phylogeny was conducted on the concatenated dataset partitioned with respect to the best models of evolution. The phylogenetic tree was visualized with MEGA 11 (Tamura et al., 2021) and the bootstrap values (bv) were added to the nodes. Bootstrap values below 50 were omitted.

The AU test was performed with IQ-TREE 2.2.0 (Minh et al., 2020; Nguyen et al., 2015). The results are available at DOI:10.5281/zenodo.10997217.

*SEM & LM analysis*

*Tryblionella apiculata* (Try946CAT and s0863) and *T. hungarica* (Try981CAT and Try986CAT), *Tryblionella gracilis*, *Tryblionella plana* var. *fennica*

The material was cleaned for microscopy using 1:1 mixtures of 96% HNO_3_ and 98% H_2_SO_4_, or domestic bleach (for s0863: described by Sato et al. (2013)), followed by thorough washing with deionized water. Aliquots were dried onto circular coverslips, which were either mounted in Naphrax for LM or attached to stubs using carbon pads for SEM. Stubs were sputtered with platinum for 70–80 s at 5 nm min^–1^ (at 25 mA) using an Emitech K575X Peltier coater and examined with a LEO Supra 55VP instrument (Zeiss/LEO, Oberkochen, Germany), usually at 5 kV, with a working distance of c. 4 mm and a tilt of 0 or 25°. LM observations were made with a Zeiss Axio Imager M2 photomicroscope or a Nikon Eclipse 90i, with Plan-Apochromat 100× objectives (N.A. 1.4) and differential interference contrast (Nomarski) optics. Photographs were taken with a DS-Ri1 (Nikon Eclipse) or an AxioCam HRc camera (Zeiss Axio Imager).

*Tryblionella navicularis* was studied in a brackish-marine sediment sample from Ferrybridge, Dorset, England, collected c. 1977 (voucher slide A108 in the diatom herbarium, Royal Botanic Garden Edinburgh). The material was cleaned with nitric and sulphuric acids, as elsewhere, and SEM observations were made in 1980 on gold-coated material (Polaron 5000 sputtering system) using a Cambridge S 150 Stereoscan, operated at 20 to 30 kV (Mann, 1982)

*Tryblionella hungarica* natural population (na. po.) from Pełczyska pond

To obtain purified benthic diatom material from the Pełczyska pond, the Żelazna-Wieczorek (2011) method was used. To obtain permanent slides, part of the purified material was applied to coverslips and, after drying, mounted in Naphrax resin (Brunel Microscopes Ltd., Chippenham, UK). The taxonomical analysis was performed with Nikon Eclipse E600 with 1000× magnification (plan oil-immersion objective 100×/1.40 Plan APO DIC H) and OPTA-TECH digital camera was used to obtain photomicrographs of cleaned frustules. To perform further analysis of the morphometry of the frustules of *T. hungarica*, one sample was selected - March, which was characterized by the largest population of this species. For morphological analysis, the purified material from the March sample was applied on metal stubs and an 8 nm gold layer was applied. SEM analysis was performed by using the Phenom Pro X (at 10 kV) scanning electron microscope at the Laboratory of Microscopy Imaging & Specialist Biological Techniques at the Faculty of Biology & Environmental Protection (University of Lodz).

*Tryblionella hungarica* SZCZ E683

Photomicrographs of living cells from the culture were obtained using Zeiss AxioScope LM with ×100 Planapochromatic objective.

To eliminate the organic components of the frustules, diatom pellets were treated with 37% hydrogen peroxide for 3 hours at 170°C. The remaining material was washed 7–10 times with distilled water. Cleaned frustules were pipetted onto coverslips, let to air dry, and then mounted on glass slides using synthetic diatom resin Naphrax^®^ for LM. For plastid images, the living diatom cultures were pipetted directly on a microscopic slide. LM images of cleaned frustules and living cells with plastids were taken using a Zeiss Axio Scope A1 (Carl Zeiss, Jena, Germany) with an oil immersion lens Zeiss Plan-Apochromat 100×/1.40 Oil M27 (Carl Zeiss, Jena, Germany) using a Canon EOS 500D camera (Canon, Tokyo, Japan) with the Canon EOS Utility software.

Morphometric and morphological analysis was performed using the same methods as for the natural population.

Capella-Gutiérrez, S., Silla-Martínez, J. M., & Gabaldón, T. (2009). trimAl: a tool for automated alignment trimming in large-scale phylogenetic analyses. *Bioinformatics*, *25*(15), 1972–3. https://doi.org/10.1093/bioinformatics/btp348

Darriba, Di., Posada, D., Kozlov, A. M., Stamatakis, A., Morel, B., & Flouri, T. (2020). ModelTest-NG: A New and Scalable Tool for the Selection of DNA and Protein Evolutionary Models. *Molecular Biology and Evolution*, *37*(1), 291–294 https://doi.org/10.1093/molbev/msz189

Flouri, T., Izquierdo-Carrasco, F., Darriba, D., Aberer, A. J., Nguyen, L. T., Minh, B. Q., Von Haeseler, A., & Stamatakis, A. 2015. The Phylogenetic Likelihood Library. *Syst Biol*, *64*(2), 356–62. https://doi.org/10.1093/sysbio/syu084.

Guillard, R. R. L. (1975). Culture of Phytoplankton for Feeding Marine Invertebrates. *Culture of Marine Invertebrate Animals*. 29–60.

Katoh, K., & Standley, D. M. (2016). A simple method to control over-alignment in the MAFFT multiple sequence alignment program. *Bioinformatics*, *32*(13), 1933–42. https://doi.org/10.1093/bioinformatics/btw108

Mann, D. G. (1982). Structure, life history and systematics of *Rhoicosphenia* (Bacillariophyta). I. The vegetative cell of *Rh. curvata*. *Journal of Phycology,* *18*(1), 162–76. https://doi.org/10.1111/j.1529-8817.1982.tb03170.x

Minh, B. Q., Schmidt, H. A., Chernomor, O., Schrempf, D., Woodhams, M. D., Von Haeseler, A., & Lanfear, R. (2020). IQ-TREE 2: New Models and Efficient Methods for Phylogenetic Inference in the Genomic Era. *Molecular Biology and Evolution,* 37(5), 1530–1534. https://doi.org/10.1093/molbev/msaa015

Nguyen, L. T., Schmidt, H. A., Von Haeseler, A., & Minh, B.Q. (2015). IQ-TREE: A Fast and Effective Stochastic Algorithm for Estimating Maximum-Likelihood Phylogenies. *Molecular Biology and Evolution*, *32*(1), 268–274. https://doi.org/10.1093/molbev/msu300

Smith, S. A., & Dunn, C. W. (2008). Phyutility: a phyloinformatics tool for trees, alignments and molecular data. *Bioinformatics,* *24*(5), 715–716. https://doi.org/10.1093/bioinformatics/btm619

Tamura, K., Stecher, G., & Kumar, S. (2021). MEGA11: Molecular Evolutionary Genetics Analysis Version 11. *Molecular Biology and Evolution*, *38*(7), 3022–7. https://doi.org/10.1093/molbev/msab120
